# Low-Density Lipoprotein Cholesterol Testing Following Myocardial Infarction Hospitalization Among Medicare Beneficiaries

**DOI:** 10.1016/j.jacadv.2023.100753

**Published:** 2023-12-08

**Authors:** Lisandro D. Colantonio, Zhixin Wang, Jenna Jones, Nafeesa N. Dhalwani, Erin D. Shannon, Cici Liu, Bethany A. Kalich, Paul Muntner, Robert S. Rosenson, Vera Bittner

**Affiliations:** a Department of Epidemiology, University of Alabama at Birmingham, Birmingham, Alabama, USA; b Center for Observational Research, Amgen Inc, Thousand Oaks, California, USA; c ICON Clinical Research Inc, Blue Bell, Pennsylvania, USA; d Amgen Inc, Thousand Oaks, California, USA; e Mount Sinai Heart, Icahn School of Medicine at Mount Sinai, New York, New York, USA; f Division of Cardiovascular Disease, Department of Medicine, University of Alabama at Birmingham, Birmingham, Alabama, USA

**Keywords:** adults, coronary artery disease, LDL, lipoproteins, medication therapy management, secondary prevention

## Abstract

**Background:**

Low-density lipoprotein cholesterol (LDL-C) is used to guide lipid-lowering therapy after a myocardial infarction (MI). Lack of LDL-C testing represents a missed opportunity for optimizing therapy and reducing cardiovascular risk.

**Objectives:**

The purpose of this study was to estimate the proportion of Medicare beneficiaries who had their LDL-C measured within 90 days following MI hospital discharge.

**Methods:**

We conducted a retrospective cohort study of Medicare beneficiaries ≥66 years of age with an MI hospitalization between 2016 and 2020. The primary analysis used data from all beneficiaries with fee-for-service coverage and pharmacy benefits (532,767 MI hospitalizations). In secondary analyses, we used data from a 5% random sample of beneficiaries with fee-for-service coverage without pharmacy benefits (10,394 MI hospitalizations), and from beneficiaries with Medicare Advantage (176,268 MI hospitalizations). The proportion of beneficiaries who had their LDL-C measured following MI hospital discharge was estimated accounting for the competing risk of death.

**Results:**

In the primary analysis (mean age 76.9 years, 84.4% non-Hispanic White), 29.9% of beneficiaries had their LDL-C measured within 90 days following MI hospital discharge. Among Hispanic, Asian, non-Hispanic White, and non-Hispanic Black beneficiaries, the 90-day postdischarge LDL-C testing was 33.8%, 32.5%, 30.0%, and 26.0%, respectively. Postdischarge LDL-C testing within 90 days was highest in the Middle Atlantic (36.4%) and lowest in the West North Central (23.4%) U.S. regions. In secondary analyses, the 90-day postdischarge LDL-C testing was 26.9% among beneficiaries with fee-for-service coverage without pharmacy benefits, and 28.6% among beneficiaries with Medicare Advantage coverage.

**Conclusions:**

LDL-C testing following MI hospital discharge among Medicare beneficiaries was low.

The measurement of low-density lipoprotein cholesterol (LDL-C) is recommended to guide lipid-lowering therapy following hospitalization for myocardial infarction (MI).^1^^,^^2^ The 2018 American Heart Association (AHA)/American College of Cardiology (ACC)/Multi-society guideline on the management of blood cholesterol recommends adults who had an MI take a statin to reduce their LDL-C by 50% or greater if they are ≤75 years of age, while at least a 30% LDL-C reduction is recommended for those >75 years of age.^1^ According to the 2022 ACC Expert Consensus Decision Pathway on Non-statin Lipid-lowering Therapy, adults with an MI who have a very high risk for recurrent atherosclerotic cardiovascular disease (ASCVD) events should consider the addition of ezetimibe and/or a proprotein convertase subtilisin/kexin type 9 monoclonal antibody (PCSK9mAb) if their LDL-C on maximally tolerated statin therapy is ≥55 mg/dL.^2^ In adults who had an MI, the measurement of LDL-C is recommended every 3 to 12 months to assess adherence and response to lipid-lowering therapy.^1^^,^^2^

Many adults with a history of MI have low adherence to lipid-lowering therapy.^3^ Also, most adults with a history of MI who have very high ASCVD risk are not taking ezetimibe or a PCSK9mAb despite having LDL-C ≥55 mg/dL.^4^^,^^5^ Lack of LDL-C testing following an MI may represent a missed opportunity to identify adults with low adherence or small LDL-C reduction following initiation of lipid-lowering therapy, and may contribute to the low use of ezetimibe and a PCSK9mAb, a potentially modifiable unmet need. The goal of the current study was to determine the proportion of Medicare beneficiaries who had their LDL-C measured within 30 days, 90 days, and 365 days following MI hospital discharge.

## Methods

We conducted a retrospective cohort study using data from Medicare beneficiaries who had an MI hospitalization. Medicare is a government program that provides health insurance for U.S. adults ≥65 years of age and adults <65 years of age with end-stage renal disease or who are disabled. About 62% of Medicare beneficiaries have fee-for-service coverage while the rest have Medicare Advantage coverage through which beneficiaries receive health care services by managed care programs.^6^ Also, 74% of Medicare beneficiaries have pharmacy benefits.^6^ For the primary analysis, we used data from all Medicare beneficiaries with fee-for-service coverage and pharmacy benefits who had an MI, hereafter referred to as the fee-for-service with pharmacy benefits cohort. To assess whether LDL-C testing varied by type of Medicare program, we conducted secondary analyses using data from a 5% random sample of Medicare beneficiaries with fee-for-service coverage with and without pharmacy benefits who had an MI, separately, hereafter referred to as the 5% fee-for-service cohort, and from MI patients with Medicare Advantage coverage in the Optum’s de-identified Clinformatics Data Mart (CDM) Database. The CDM Database is one of the largest data sets of beneficiaries with Medicare Advantage coverage including health insurance for physician, hospital, and prescription drug services. The Institutional Review Board at the University of Alabama at Birmingham approved the study and waived the requirement to obtain informed consent.

### Study sample

For the primary analysis, we included patients in the fee-for-service with pharmacy benefits cohort who were ≥66 years of age on the date of their MI hospital discharge between January 1, 2016, and December 31, 2020. An MI hospitalization was defined by an inpatient claim with an overnight stay and <30 days of duration with an International Classification of Diseases-10th Revision code of I21.xxx or I22.xxx in any discharge diagnosis position. We excluded patients <66 years of age on the day they were discharged from the hospital for MI, <65 years of age 365 days before their MI hospital discharge date, as this group is not representative of the general population. We restricted the analysis to patients who had continuous fee-for-service inpatient, outpatient, and pharmacy coverage and were living in the United States for 365 days before their MI hospital discharge and were discharged alive. We further restricted the analysis to patients who were alive and had Medicare fee-for-service inpatient, outpatient, and pharmacy coverage for 30 days following their MI hospital discharge to determine postdischarge characteristics. We excluded patients who were admitted to a skilled nursing facility within 30 days after their MI hospital discharge. After these inclusion/exclusion criteria were applied, 532,767 MI hospitalizations were included in the primary analysis (Supplemental Figure 1). For the secondary analyses of the 5% fee-for-service cohort, there were 21,690 MI hospitalizations among patients with continuous pharmacy coverage from 365 days before through 30 days after their MI hospital discharge, and 10,394 MI hospitalizations among patients without continuous pharmacy coverage that met the inclusion/exclusion criteria (Supplemental Figure 2). For the secondary analysis using the CDM Database, 176,268 MI hospitalizations met the inclusion/exclusion criteria (Supplemental Figure 3). Supplemental Figure 4 shows a schematic for the study.

### LDL-C testing

We used outpatient claims to identify LDL-C tests following each patient’s MI hospital discharge. LDL-C testing within 90 days was selected a priori as the primary outcome, with LDL-C testing within 30 and 365 days following MI hospital discharge as secondary outcomes. An LDL-C test was defined by a Current Procedural Terminology (CPT) code of 83721 (direct LDL-C measurement) or 80061 (a lipid panel of total cholesterol, high-density lipoprotein cholesterol, and triglycerides, through which LDL-C can be calculated).^7^^,^^8^ To determine whether this definition underestimates the frequency of LDL-C testing, we conducted a sensitivity analysis assessing lipid testing defined using the broader set of CPT codes shown in Supplemental Table 1. This definition of lipid testing included lipid measurements which may indicate that LDL-C was known, eg, lipoprotein(a).

### Patient characteristics

We used administrative data to determine each patient’s age on their MI hospital discharge date, sex, race/ethnicity, place of residence, and 2 markers of low socioeconomic status, dual Medicare-Medicaid eligibility, and receipt of a low-income subsidy for prescription medications. We used all available claims before each patient’s MI hospital discharge to define comorbid conditions including diabetes, chronic kidney disease, heart failure, history of stroke, and history of lower extremity artery disease. We used claims before each patient’s MI hospital admission date to define a history of a recent acute coronary syndrome (in the prior 12 months) and a history of coronary heart disease. We used procedure codes during the MI hospitalization to identify if a coronary revascularization procedure occurred. We used pharmacy claims from the 90 days before each patient’s MI hospital admission through 7 days after each patient’s MI hospital discharge to determine the use of a statin, ezetimibe, and PCSK9mAb. We used claims within 30 days after each patient’s MI hospital discharge to identify outpatient cardiologist and primary care physician visits, rehospitalizations, recurrent MIs, revascularization procedures, and cardiac rehabilitation sessions. For patients with an LDL-C test within 30 days of their MI hospital discharge, we considered outpatient cardiologist and primary care physician visits, rehospitalizations, recurrent MIs, revascularization procedures, and cardiac rehabilitation sessions to have occurred only if these preceded or may have led to the LDL-C test. Definitions for patient characteristics are shown in Supplemental Table 2.

### Statistical analysis

We calculated summary statistics for characteristics of patients in the fee-for-service with pharmacy benefits cohort, overall, and among those with and without an LDL-C test within 90 days following MI hospital discharge. We calculated the cumulative incidence of an LDL-C test at 90 days following hospital discharge for MI. We censored patients when they lost fee-for-service inpatient or outpatient coverage. We accounted for the competing risk of death using the Fine and Grey approach to not overestimate the absolute cumulative incidence of an LDL-C test.^9^ Cumulative incidence estimates were generated for the overall population and for subgroups defined by each of the patient characteristics included in the analysis (Supplemental Table 2). We used bootstrapping techniques to calculate ratios and 95% CIs for the cumulative incidence of an LDL-C test at 90 days following MI hospital discharge associated with patient characteristics including adjustment for all characteristics simultaneously.^10^ The analyses described above were repeated to estimate the cumulative incidence of an LDL-C test at 30 days and 365 days following MI hospital discharge. In a sensitivity analysis, we calculated the cumulative incidence of a lipid test, rather than an LDL-C test, at 90 days, 30 days, and 365 days following MI hospital discharge. Measuring LDL-C within 30 days following MI hospital discharge may not be appropriate to assess the response to treatment changes.^2^ In another sensitivity analysis, we measured the cumulative incidence of an LDL-C test after MI hospital discharge excluding tests in the initial 30 days postdischarge.

In secondary analyses, we calculated characteristics and the cumulative incidence of an LDL-C test at 90 days, 30 days, and 365 days following MI hospital discharge among patients in the 5% fee-for-service cohort with and without continuous pharmacy benefits, separately, and in the CDM Database (ie, among Medicare beneficiaries with managed care). Among patients in the 5% fee-for-service cohort, we calculated the ratios and 95% CIs for the cumulative incidence of an LDL-C test at 90 days, 30 days, and 365 days following MI hospital discharge, separately, associated with not having continuous pharmacy benefits, adjusting for patient characteristics. Statistical analyses were conducted in SAS, except for bootstrapping analyses which were conducted in R.

## Results

The mean age of patients in the fee-for-service with pharmacy benefits cohort was 76.9 years, 52.9% were male, and 84.4% were non-Hispanic White. Patients with an LDL-C test within 90 days following hospital discharge for MI were younger and more likely to be male vs their counterparts without an LDL-C test (Table 1). Supplemental Tables 3 and 4 show characteristics of patients with and without an LDL-C test within 30 days and 365 days following MI hospital discharge, respectively.

**Table 1:** Characteristic of Patients in the Fee-For-Service With Pharmacy Benefits Cohort, Overall and Among Those With and Without an LDL-C Test Within 90 Days Following MI Hospital Discharge

	Overall (N = 532,767)	LDL-C Test Within 90 d Following MI Hospital Discharge	
		No (n = 375,659)	Yes (n = 157,108)
Calendar year of the MI hospital discharge			
2016	96,344 (18.1)	66,122 (17.6)	30,222 (19.2)
2017	104,630 (19.6)	72,802 (19.4)	31,828 (20.3)
2018	110,484 (20.7)	77,090 (20.5)	33,394 (21.3)
2019	116,509 (21.9)	81,583 (21.7)	34,926 (22.2)
2020	104,800 (19.7)	78,062 (20.8)	26,738 (17.0)
Age			
66-75 y	258,741 (48.6)	176,581 (47.0)	82,160 (52.3)
≥76 y	274,026 (51.4)	199,078 (53.0)	74,948 (47.7)
Male, n (%)	281,843 (52.9)	194,597 (51.8)	87,246 (55.5)
Race/ethnicity			
Non-Hispanic White	449,491 (84.4)	316,434 (84.2)	133,057 (84.7)
Non-Hispanic Black	44,602 (8.4)	33,167 (8.8)	11,435 (7.3)
Asian	10,566 (2.0)	7,183 (1.9)	3,383 (2.2)
Hispanic	10,485 (2.0)	6,987 (1.9)	3,498 (2.2)
Other	17,623 (3.3)	11,888 (3.2)	5,735 (3.7)
Geographic region of residence			
New England	34,985 (6.6)	25,119 (6.7)	9,866 (6.3)
West South Central	58,789 (11.0)	41,628 (11.1)	17,161 (10.9)
Mountain	27,073 (5.1)	19,645 (5.2)	7,428 (4.7)
East South Central	39,734 (7.5)	27,986 (7.4)	11,748 (7.5)
Middle Atlantic	72,518 (13.6)	46,444 (12.4)	26,074 (16.6)
South Atlantic	109,802 (20.6)	74,789 (19.9)	35,013 (22.3)
West North Central	37,787 (7.1)	29,083 (7.7)	8,704 (5.5)
East North Central	90,772 (17.0)	67,977 (18.1)	22,795 (14.5)
Pacific	61,307 (11.5)	42,988 (11.4)	18,319 (11.7)
Dual eligibility/low-income subsidy for medications	143,861 (27.0)	105,737 (28.1)	38,124 (24.3)
Diabetes	254,592 (47.8)	173,226 (46.1)	81,366 (51.8)
Chronic kidney disease	305,478 (57.3)	219,326 (58.4)	86,152 (54.8)
Heart failure	280,715 (52.7)	204,652 (54.5)	76,063 (48.4)
History of stroke	41,150 (7.7)	30,024 (8.0)	11,126 (7.1)
History of lower extremity artery disease	116,322 (21.8)	83,217 (22.2)	33,105 (21.1)
Recent ACS	76,404 (14.3)	55,838 (14.9)	20,566 (13.1)
History of CHD	320,979 (60.2)	228,249 (60.8)	92,730 (59.0)
Coronary revascularization during the MI hospitalization	288,073 (54.1)	189,677 (50.5)	98,396 (62.6)
Medication use^a^			
Statin use			
Intensity			
None	131,738 (24.7)	100,025 (26.6)	31,713 (20.2)
Low-/moderate-intensity therapy	155,773 (29.2)	109,952 (29.3)	45,821 (29.2)
High-intensity therapy	245,256 (46.0)	165,682 (44.1)	79,574 (50.6)
Initiation	124,866 (23.4)	83,683 (22.3)	41,183 (26.2)
Up-titration	38,826 (7.3)	24,644 (6.6)	14,182 (9.0)
Ezetimibe use			
Prevalent use	15,406 (2.9)	9,678 (2.6)	5,728 (3.6)
Initiation	3,342 (0.6)	1,955 (0.5)	1,387 (0.9)
PCSK9mAb^b^ use	1,980 (0.4)	1,073 (0.3)	907 (0.6)
Patient characteristics defined using claims in the 30 d after each patient’s MI hospital discharge date^c^			
Cardiologist outpatient visits	194,072 (36.4)	131,225 (34.9)	62,847 (40.0)
Primary care physician outpatient visits	253,283 (47.5)	178,258 (47.5)	75,025 (47.8)
Rehospitalization	77,592 (14.6)	61,239 (16.3)	16,353 (10.4)
Recurrent MI	25,227 (4.7)	19,350 (5.2)	5,877 (3.7)
Coronary revascularization procedure	114,062 (21.4)	77,408 (20.6)	36,654 (23.3)
Cardiac rehabilitation	40,959 (7.7)	26,992 (7.2)	13,967 (8.9)

Values are n (%). The definition of LDL-C test includes an outpatient claim with a Current Procedural Terminology code of 83721 or 80061.

ACS = acute coronary syndrome; CHD = coronary heart disease; LDL-C = low-density lipoprotein cholesterol; MI = myocardial infarction; PCSK9mAb = proprotein convertase subtilisin/kexin type 9 monoclonal antibody.

a Medication use was defined using pharmacy claims in the 90 days prior to each patient’s MI hospital admission date and within 7 days after each patient’s MI hospital discharge date. Definitions of medication use are provided in Supplemental Table 2.

b PCSK9mAbs were referred as proprotein convertase subtilisin/kexin type 9 inhibitors in the 2018 American Heart Association (AHA)/American College of Cardiology (ACC)/Multi-society guideline on the management of blood cholesterol.^1^

c For patients with an LDL-C test within 30 days after their MI hospital discharge date, patient characteristics in the 30 days after their MI hospital discharge date were considered to be present only if these preceded or may have led to the LDL-C test. Specifically, we considered cardiologist care and primary care ambulatory visits to have occurred only if patients did not have an LDL-C test between their MI hospital discharge date and 3 days before their earliest cardiologist or primary care physician visits, respectively. We included outpatient cardiologist and primary care physician visits up to 3 days after an LDL-C test as this may have been ordered in advance of the visit. We considered rehospitalization, recurrent MI, coronary revascularization procedure, and cardiac rehabilitation to have occurred only if patients did not have an LDL-C test between their MI hospital discharge date and the date of their earliest rehospitalization, recurrent MI, coronary revascularization procedure, or cardiac rehabilitation session, respectively.

**Figure 1:**
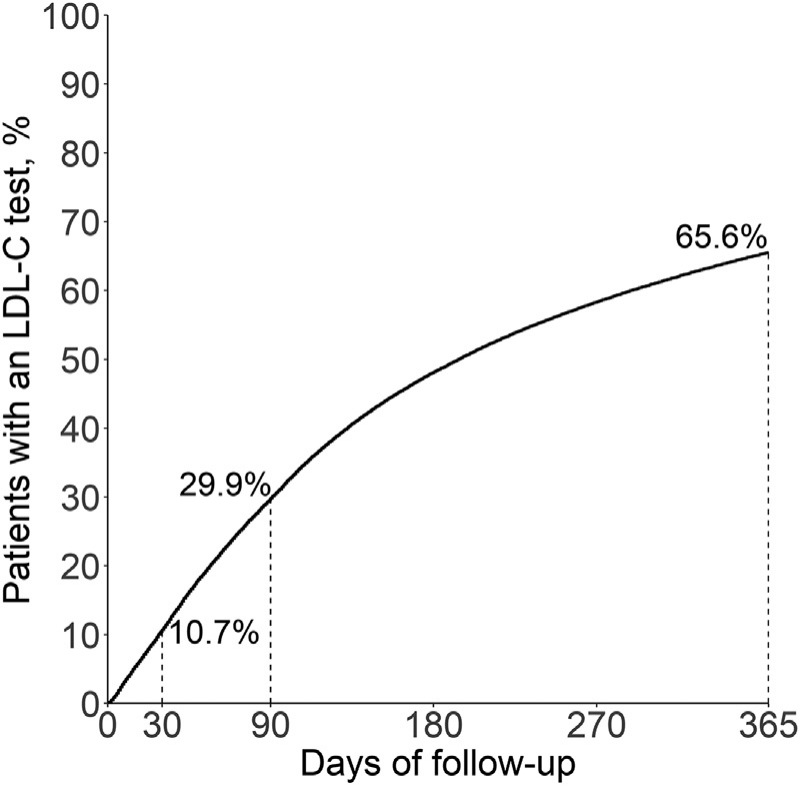
**Cumulative Incidence of an LDL-C Test Following Hospital Discharge for MI in the Fee-For-Service With Pharmacy Benefits Cohort** The definition of LDL-C test includes an outpatient claim with a Current Procedural Terminology code of 83721 or 80061. LDL-C = low-density lipoprotein cholesterol; MI = myocardial infarction.

**Figure 2:**
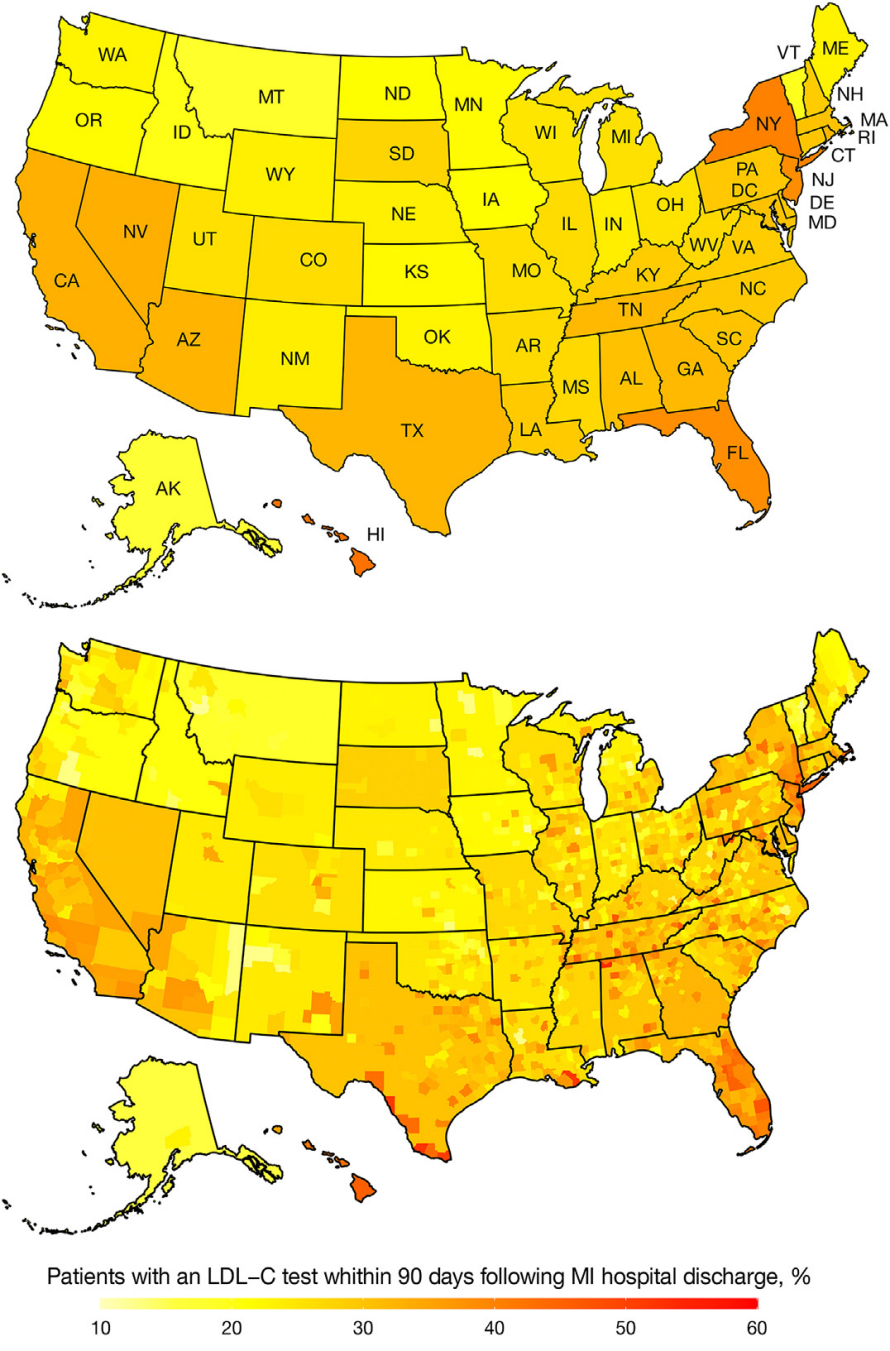
Cumulative Incidence of an LDL-C Test at 90 Days Following MI Hospital Discharge by Place of Residence in the Fee-for-Service With Pharmacy Benefits Cohort The top panel shows the cumulative incidence of an LDL-C test at 90 days following MI hospital discharge by state. The bottom panel shows the cumulative incidence of an LDL-C test at 90 days following MI hospital discharge by county of residence. The definition of LDL-C test includes an outpatient claim with a Current Procedural Terminology code of 83721 or 80061. LDL-C = low-density lipoprotein cholesterol; MI = myocardial infarction.

The cumulative incidence of an LDL-C test at 30 days, 90 days, and 365 days following MI hospital discharge was 10.7%, 29.9%, and 65.6%, respectively (Figure 1). The cumulative incidence was lower in 2020 vs 2016, and among older adults, females, those of non-Hispanic Black race vs other race/ethnicity subgroups, living in the West North Central vs other U.S. regions, with dual eligibility/low-income subsidy, chronic kidney disease, a history of stroke, lower extremity artery disease, recent acute coronary syndrome, or coronary heart disease (Table 2). Figure 2 shows the cumulative incidence of an LDL-C test at 90 days following MI hospital discharge by state and county of residence. Supplemental Table 5 shows the cumulative incidence of an LDL-C test at 30, 90, and 365 days following MI hospital discharge by state.

**Table 2:** Cumulative Incidence of an LDL-C Test by Patient Characteristics in the Fee-For-Service With Pharmacy Benefits Cohort

	Cumulative Incidence, %		
	30 d	90 d	365 d
Calendar year of the MI hospital discharge			
2016	11.0	31.4	67.3
2017	10.8	30.5	66.7
2018	11.0	30.3	66.3
2019	10.7	30.0	64.5
2020	10.2	27.1	62.4
Age			
66-75 y	11.3	32.2	69.6
≥76 y	10.2	27.7	61.9
Female	10.1	28.2	63.6
Male	11.3	31.4	67.4
Race/ethnicity			
Non-Hispanic White	10.6	30.0	66.3
Non-Hispanic Black	10.0	26.0	57.8
Asian	12.9	32.5	65.7
Hispanic	13.6	33.8	66.8
Other	12.4	33.0	66.3
Geographic region of residence			
New England	10.0	28.6	63.2
West South Central	10.7	29.6	65.6
Mountain	9.8	27.8	61.6
East South Central	10.7	29.9	66.6
Middle Atlantic	14.2	36.4	71.1
South Atlantic	11.3	32.3	69.4
West North Central	7.9	23.4	60.3
East North Central	8.4	25.5	62.2
Pacific	11.6	30.3	63.1
Dual eligibility/low-income subsidy for medications			
Yes	10.3	26.9	58.7
No	10.9	31.0	68.1
Diabetes			
Yes	12.1	32.4	68.3
No	9.5	27.6	63.1
Chronic kidney disease			
Yes	10.9	28.6	62.1
No	10.5	31.6	70.3
Heart failure			
Yes	10.5	27.5	60.3
No	11.0	32.6	71.5
History of stroke			
Yes	9.8	27.4	60.6
No	10.8	30.1	66.0
History of lower extremity artery disease			
Yes	10.9	28.8	61.9
No	10.7	30.2	66.7
Recent ACS			
Yes	10.5	27.3	59.6
No	10.8	30.3	66.6
History of CHD			
Yes	10.9	29.3	64.6
No	10.4	30.8	67.1
Coronary revascularization during the MI hospitalization			
Yes	12.1	34.5	72.8
No	9.1	24.4	57.0
Medication use^a^			
Statin use			
Intensity			
None	9.0	24.4	55.7
Low-/moderate-intensity therapy	10.7	29.8	66.7
High-intensity therapy	11.6	32.9	70.2
Initiation			
Yes	11.2	33.4	69.6
No	10.6	28.8	64.4
Up-titration			
Yes	12.8	37.0	76.1
No	10.6	29.3	64.8
Ezetimibe use			
Prevalent use			
Yes	14.1	37.7	76.2
No	10.6	29.6	65.3
Initiation			
Yes	14.8	42.2	79.7
No	10.7	29.8	65.5
PCSK9mAb^b^ use			
Yes	18.1	47.0	85.7
No	10.7	29.8	65.6
Patient characteristics defined using claims in the 30 d after each patient’s MI hospital discharge date^c^			
Cardiologist outpatient visits			
Yes	10.4	32.7	71.4
No	10.9	28.2	62.2
Primary care physician outpatient visits			
Yes	10.8	30.0	66.7
No	10.6	29.8	64.7
Rehospitalization			
Yes	4.8	21.5	54.2
No	11.7	31.3	67.6
Recurrent MI			
Yes	5.6	23.7	57.8
No	11.0	30.2	66.0
Coronary revascularization procedure			
Yes	10.2	32.5	71.9
No	10.9	29.2	63.9
Cardiac rehabilitation			
Yes	5.7	34.5	79.7
No	11.1	29.5	64.4

Values are %. The definition of LDL-C test includes an outpatient claim with a Current Procedural Terminology code of 83721 or 80061.

ACS = acute coronary syndrome; CHD = coronary heart disease; LDL-C = low-density lipoprotein cholesterol; MI = myocardial infarction; PCSK9mAb = proprotein convertase subtilisin/kexin type 9 monoclonal antibody.

a Medication use was defined using pharmacy claims in the 90 days prior to each patient’s MI hospital admission date through 7 days after each patient’s MI hospital discharge date. Definitions of medication use are provided in Supplemental Table 2.

b PCSK9mAbs were referred as proprotein convertase subtilisin/kexin type 9 inhibitors in the 2018 American Heart Association (AHA)/American College of Cardiology (ACC)/Multi-society guideline on the management of blood cholesterol.^1^

c For patients with an LDL-C test within 30 days after their MI hospital discharge date, patient characteristics in the 30 days after their MI hospital discharge date were considered to be present only if these preceded or may have led to the LDL-C test. Specifically, we considered cardiologist care and primary care ambulatory visits to have occurred only if patients did not have an LDL-C test between their MI hospital discharge date and 3 days before their earliest cardiologist or primary care physician visits, respectively. We included outpatient cardiologist and primary care physician visits up to 3 days after an LDL-C test as this may have been ordered in advance of the visit. We considered rehospitalization, recurrent MI, coronary revascularization procedure, and cardiac rehabilitation to have occurred only if patients did not have an LDL-C test between their MI hospital discharge date and the date of their earliest rehospitalization, recurrent MI, coronary revascularization procedure, or cardiac rehabilitation session, respectively.

After multivariable adjustment, having an LDL-C test within 90 days following MI hospital discharge was less likely in 2020 vs 2016, and among patients ≥76 years vs 66 to 75 years of age, of non-Hispanic Black vs non-Hispanic White race/ethnicity, living in the West North Central, East North Central, and Mountain regions vs the New England region, with dual eligibility/low-income subsidy, heart failure, and in those with a rehospitalization in the 30 days following MI hospital discharge (Table 3). Having an LDL-C test within 90 days following MI hospital discharge was more likely in patients of Hispanic or Asian vs non-Hispanic White race/ethnicity, living in the Middle Atlantic or South Atlantic regions vs the New England region, with diabetes or a coronary revascularization procedure during their MI, taking high-intensity statin therapy or who initiated or up-titrated this medication, those taking or initiating ezetimibe, and those taking a PCSK9mAb. Supplemental Table 6 shows patient characteristics associated with having an LDL-C test within 30 days and 365 days following MI hospital discharge. In sensitivity analyses, the cumulative incidence of lipid testing (rather than LDL-C testing) at 30, 90, and 365 days following MI hospital discharge was 11.0%, 30.3%, and 66.2%, respectively (Supplemental Figure 5). Excluding tests within 30 days following MI hospital discharge, the cumulative incidence of an LDL-C test at 30, 60, 90, 120, 365, and 395 days was 0%, 11.8%, 21.5%, 30.6%, 62.8%, and 64.9%, respectively (Supplemental Figure 6).

**Table 3:** Patient Characteristic Associated With Having an LDL-C Test Within 90 Days Following Hospital Discharge for MI in the Fee-For-Service With Pharmacy Benefits Cohort

	Cumulative Incidence at 90 d Following MI Hospital Discharge
Calendar year of the MI hospital discharge	
2016	1.00 (reference)
2017	0.98 (0.97–0.99)
2018	0.98 (0.97–1.00)
2019	0.98 (0.97–0.99)
2020	0.91 (0.90–0.92)
Age	
66-75 y	1.00 (reference)
≥76 y	0.91 (0.91–0.92)
Female	1.00 (reference)
Male	1.02 (1.01–1.03)
Race/ethnicity	
Non-Hispanic White	1.00 (reference)
Non-Hispanic Black	0.96 (0.95–0.98)
Asian	1.12 (1.09–1.15)
Hispanic	1.19 (1.16–1.22)
Other	1.07 (1.04–1.09)
Geographic region of residence	
New England	1.00 (reference)
West South Central	1.03 (1.01–1.05)
Mountain	0.92 (0.90–0.94)
East South Central	1.05 (1.03–1.07)
Middle Atlantic	1.25 (1.22–1.27)
South Atlantic	1.11 (1.09–1.13)
West North Central	0.80 (0.78–0.82)
East North Central	0.89 (0.87–0.91)
Pacific	1.05 (1.03–1.07)
Dual eligibility/Low-income subsidy for medications	0.89 (0.88–0.90)
Diabetes	1.20 (1.19–1.21)
Chronic kidney disease	0.99 (0.98–1.00)
Heart failure	0.92 (0.91–0.92)
History of stroke	0.97 (0.96–0.99)
History of lower extremity artery disease	1.00 (0.99–1.01)
Recent ACS	0.97 (0.96–0.98)
History of CHD	0.96 (0.95–0.97)
Coronary revascularization during the MI hospitalization	1.25 (1.24–1.27)
Medication use^a^	
Statin use	
Intensity	
None	1.00 (reference)
Low-/moderate-intensity therapy	1.15 (1.13–1.16)
High-intensity therapy	1.17 (1.15–1.18)
Initiation (vs no initiation)	1.04 (1.03–1.06)
Up-titration (vs no up-titration)	1.13 (1.11–1.14)
Ezetimibe use	
Prevalent use (vs no prevalent use)	1.23 (1.20–1.25)
Initiation (vs no initiation)	1.35 (1.30–1.41)
PCSK9mAb^b^ use	1.54 (1.47–1.61)
Patient characteristics defined using claims in the 30 d after each patient’s MI hospital discharge date^c^	
Cardiologist outpatient visits	1.06 (1.05–1.07)
Primary care physician outpatient visits	1.01 (1.00–1.02)
Rehospitalization	0.73 (0.71–0.74)
Recurrent MI	1.03 (1.02–1.05)
Coronary revascularization procedures	1.01 (1.00–1.02)
Cardiac rehabilitation	1.02 (1.00–1.04)

Values are HR (95% CI). The definition of LDL-C test includes an outpatient claim with a Current Procedural Terminology code of 83721 or 80061.

Ratios include adjustment for all patient characteristics simultaneously.

ACS = acute coronary syndrome; CHD = coronary heart disease; LDL-C = low-density lipoprotein cholesterol; MI = myocardial infarction; PCSK9mAb = proprotein convertase subtilisin/kexin type 9 monoclonal antibody.

a Medication use was defined using pharmacy claims in the 90 days prior to each patient’s MI hospital admission date through 7 days after each patient’s MI hospital discharge date. Definitions of medication use are provided in Supplemental Table 2.

b PCSK9mAbs were referred as proprotein convertase subtilisin/kexin type 9 inhibitors in the 2018 American Heart Association (AHA)/American College of Cardiology (ACC)/Multi-society guideline on the management of blood cholesterol.^1^

c For patients with an LDL-C test within 30 days after their MI hospital discharge date, patient characteristics in the 30 days after their MI hospital discharge date were considered to be present only if these preceded or may have led to the LDL-C test. Specifically, we considered cardiologist care and primary care ambulatory visits to have occurred only if patients did not have an LDL-C test between their MI hospital discharge date and 3 days before their earliest cardiologist or primary care physician visits, respectively. We included outpatient cardiologist and primary care physician visits up to 3 days after an LDL-C test as this may have been ordered in advance of the visit. We considered rehospitalization, recurrent MI, coronary revascularization procedure, and cardiac rehabilitation to have occurred only if patients did not have an LDL-C test between their MI hospital discharge date and the date of their earliest rehospitalization, recurrent MI, coronary revascularization procedure, or cardiac rehabilitation session, respectively.

### Secondary analyses

Supplemental Table 7 shows characteristics of Medicare beneficiaries in the 5% fee-for-service cohort who had and did not have continuous pharmacy benefits from 365 days prior to through 30 days after their MI hospital discharge. The cumulative incidence of an LDL-C test at 30 days, 90 days, and 365 days following MI hospital discharge was lower in Medicare beneficiaries who did not have vs who had continuous pharmacy benefits (Table 4, Supplemental Figure 7). After multivariable adjustment, patients without vs with continuous pharmacy benefits were less likely to have an LDL-C test at 30 days (HR: 0.92; 95% CI: 0.86-0.98), 90 days (HR: 0.86; 95% CI: 0.83-0.90), and 365 days (HR: 0.94; 95% CI: 0.93-0.95) following MI hospital discharge. Supplemental Table 8 shows the characteristics of Medicare beneficiaries in the CDM Database. Among patients in the CDM Database, the cumulative incidence of an LDL-C test at 30 days, 90 days, and 365 days following MI hospital discharge was 10.2%, 28.6%, and 60.9%, respectively.

**Table 4:** LDL-C Testing Following MI Hospital Discharge Among Medicare Beneficiaries in the 5% Fee-For-Service Cohort (Secondary Analysis)

Follow-Up Time	Patients With Continuous Pharmacy Benefits From 365 d Prior to Through 30 d After Their MI Hospital Discharge^b^	
	No (n = 10,394)	Yes (n = 21,690)
30 d following MI hospital discharge		
Cumulative incidence, %	10.3	11.1
Multivariable-adjusted HR (95% CI)^a^	0.92 (0.86-0.98)	1.00 (ref)
90 d following MI hospital discharge		
Cumulative incidence, %	26.9	30.8
Multivariable-adjusted HR (95% CI)^a^	0.86 (0.83-0.90)	1.00 (ref)
365 d following MI hospital discharge		
Cumulative incidence, %	59.8	66.6
Multivariable-adjusted HR (95% CI)^a^	0.94 (0.93-0.95)	1.00 (ref)

LDL-C = low-density lipoprotein cholesterol; MI = myocardial infarction.

a Ratios include adjustment for calendar year of the MI hospital discharge, age, sex, geographic region of residence, diabetes, chronic kidney disease, heart failure, history of stroke, lower extremity artery disease event, recent acute coronary syndrome, coronary heart disease, coronary revascularization procedure during the MI hospitalization, and characteristics measured in the 30 days following MI hospital discharge, including cardiologist care visit, primary care ambulatory visit, rehospitalization, recurrent MI, coronary revascularization procedure, and cardiac rehabilitation. The analysis did not include adjustment for low-income subsidy for prescription medications and use of statin, ezetimibe, and proprotein convertase subtilisin/kexin type 9 monoclonal antibody as these may not be detected in patients without pharmacy benefits.

b Having continuous pharmacy benefits was defined as having pharmacy coverage from 365 days prior to through 30 days after each patient’s MI hospital discharge date.

## Discussion

In the current analysis of Medicare beneficiaries ≥66 years of age with an MI hospitalization, a small proportion had an LDL-C test in the 90 days following hospital discharge. There were disparities in LDL-C testing following MI hospital discharge by race/ethnicity, geographic region of residence, and type of Medicare program (Central Illustration). Having an LDL-C test following MI hospitalization may be influenced by patient characteristics and clinical management practices.

**Central Illustration:**
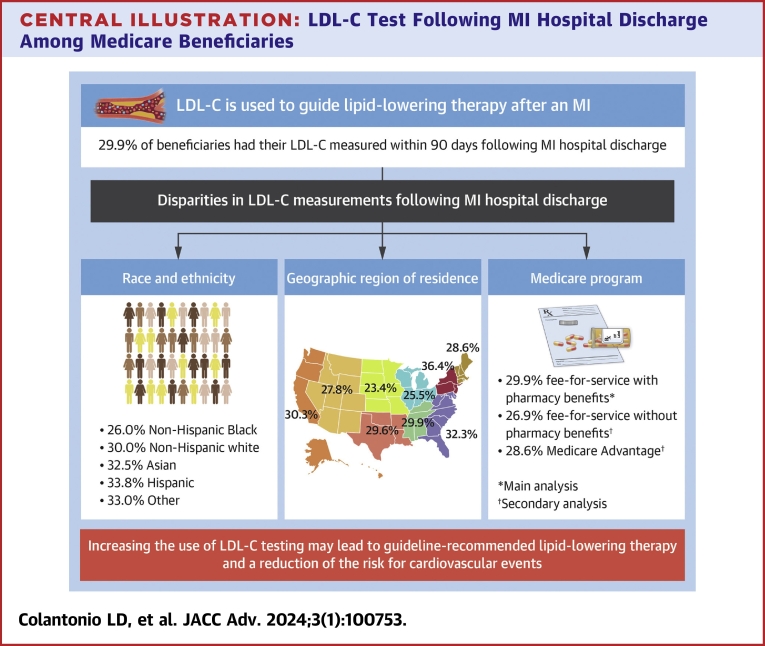
**LDL-C Test Following MI Hospital Discharge Among Medicare Beneficiaries** Some icons were adapted from SVG REPO (https://www.svgrepo.com/). FFS = fee-for-service; LDL-C = low-density lipoprotein cholesterol; MI = myocardial infarction.

Lipid testing can overcome clinical inertia and is associated with lipid-lowering medication use and initiation,^11^, ^12^, ^13^ higher adherence to lipid-lowering medication,^14^^,^^15^ and lipid-lowering treatment intensification.^12^^,^^15^, ^16^, ^17^ In a prior analysis of veterans with a history of ASCVD who had a primary care physician visit at the Veterans Health Administration (VA), those with a lipid panel had a higher likelihood of lipid-lowering treatment intensification within 90 days vs their counterparts without a lipid panel (9.3% vs 5.4%, respectively, *P* < 0.001).^16^ In another study, the percentage of VA patients with a lipid test within 90 days and 365 days following an MI hospitalization or elective coronary revascularization was 37.0% and 81.7%, respectively.^12^ Patients included in the latter VA analysis were younger (mean age 69.0 years) compared to those included in the current study (mean age 76.9 years), which may contribute to explaining the modestly lower use of lipid testing among Medicare beneficiaries.^12^ Increasing the use of LDL-C testing among Medicare beneficiaries following an MI may lead to guideline-recommended lipid-lowering therapy and a reduction of the risk for recurrent cardiovascular events.

Several factors were associated with LDL-C testing in the current study. LDL-C testing following MI was lower in non-Hispanic Black vs non-Hispanic White beneficiaries and among those with markers of low socioeconomic status. LDL-C testing was also less common in older beneficiaries and those with a history of heart failure, which may be explained by their lower life expectancy and a perceived low benefit of rigorous cholesterol management in these populations. Adults with diabetes or who received a coronary revascularization procedure during their MI hospitalization were more likely to receive LDL-C testing. These associations may be explained by a perceived need for more intensive lipid-lowering therapy in these individuals. Actions aimed at increasing the use of LDL-C testing among Medicare beneficiaries who had an MI should reach vulnerable populations including those of non-Hispanic Black race/ethnicity and those with low socioeconomic status to prevent widening health inequities.

We have documented substantial geographic disparities in the use of high-intensity statin therapy following MI among Medicare beneficiaries.^18^ In the current analysis, LDL-C testing at 90 days following MI hospital discharge was highest in the Middle Atlantic and lowest in the West North Central U.S. regions. Geographic disparities in LDL-C testing and high-intensity statin therapy use may be explained by differences in the adoption of clinical recommendations across U.S. regions. The 2013 ACC/AHA guideline on the management of blood cholesterol recommended the use of fixed-dose statin therapy without specific LDL-C treatment goals.^19^ Full implementation of this guideline was expected to increase high-intensity statin use with a concomitant decline in LDL-C testing.^20^ Although the 2013 ACC/AHA blood cholesterol guideline included LDL-C testing as a Class 1A recommendation, an unintentional effect of this guideline was the misconception that documenting the response and adherence to statin therapy through LDL-C testing was not needed.^14^^,^^21^^,^^22^ In a prior study, LDL-C testing among patients with commercial health insurance and a history of ASCVD who were taking a statin was 270 and 254 per 1,000 person-calendar-year-quarters in 2007 and 2016, respectively.^23^ Results from the current study also suggest that the use of LDL-C tests may have declined during the COVID-19 pandemic.^24^^,^^25^ Despite national improvements in high-intensity statin use,^26^ many adults with a history of MI may have low adherence to this medication or may benefit from adding ezetimibe or a PCSK9mAb.^3^, ^4^, ^5^^,^^27^ Interventions aimed at improving blood cholesterol management including LDL-C testing may need to consider differences in unmet needs and clinical practice across regions.

Medicare provides health insurance through different types of programs, and the characteristics of individuals enrolled in these programs may vary. Medicare beneficiaries without pharmacy benefits are more likely to have low income, less than high school education, and not be married, vs those with pharmacy benefits.^6^ Beneficiaries with Medicare Advantage are more likely to have low income or be older or non-Hispanic Black or Hispanic vs those with Medicare fee-for-service coverage.^6^ In the current analysis, LDL-C testing at 30, 90, and 365 days following MI hospital discharge occurred less frequently in Medicare beneficiaries with fee-for-service coverage who did not have pharmacy benefits vs their counterparts with pharmacy benefits. LDL-C testing at 365 days following MI hospital discharge also appeared to be lower among beneficiaries with Medicare Advantage in the CDM Database vs Medicare beneficiaries with fee-for-service coverage and pharmacy benefits.

After the publication of the 2013 blood cholesterol guideline, the 2015 AHA/ACC Report on Performance Measures removed LDL-C testing as a quality metric in adults with a prior MI.^28^^,^^29^ The same action was taken by other national quality organizations and payers including Medicare.^22^ Re-establishing LDL-C testing as a quality metric may be a simple, cheap intervention to improve guideline-recommended blood cholesterol management.^14^^,^^15^^,^^22^

### Strengths and limitations

The current analysis has several strengths. We used data from a large number of Medicare beneficiaries with an MI from all U.S. states and with different types of insurance programs. Also, we analyzed several beneficiary characteristics and comorbidities. The current study has known and potential limitations. LDL-C values are not available in Medicare claims. However, guidelines recommend that all patients with a prior MI have their LDL-C measured at a minimum once every 12 months (365 days) regardless of prior LDL-C levels and current lipid-lowering therapy.^1^^,^^2^ The validity of the CPT-based algorithm used to identify LDL-C tests in Medicare claims is unknown. Also, only claims for ambulatory LDL-C tests submitted to Medicare were available for the current study. We cannot exclude that some Medicare beneficiaries may have had an LDL-C test that was not submitted to Medicare (eg, if this was done in the VA or paid through supplementary insurance), which may have resulted in an underestimation of LDL-C testing. Also, we cannot determine from claims whether LDL-C testing was performed to assess treatment or for some other reasons. Counting all tests performed may overestimate the use of LDL-C testing to guide lipid-lowering therapy. We used data from Medicare beneficiaries ≥66 years of age. The results may not be generalizable to younger Medicare beneficiaries or adults without Medicare health insurance. For the secondary analyses, we used data from a 5% random sample of beneficiaries with Medicare fee-for-service coverage. LDL-C testing following MI hospital discharge among beneficiaries with continuous pharmacy benefits in the secondary analysis was consistent with the primary analysis, supporting the validity of using a 5% random sample. Also, we used data from beneficiaries with Medicare Advantage in the CDM Database. Therefore, results may not be generalizable to all beneficiaries with Medicare Advantage coverage. Prior studies have shown that LDL-C testing leads to improvements in lipid-lowering medication use and adherence.^11^, ^12^, ^13^, ^14^, ^15^, ^16^, ^17^ This was not possible in the current analysis due to not having LDL-C values.

## Conclusions

A low proportion of Medicare beneficiaries had their LDL-C measured following MI hospital discharge, with disparities by race/ethnicity, geographic region of residence, and type of Medicare program. This represents a missed opportunity to identify patients with low adherence and response to lipid-lowering medication, and to optimize treatment to reduce the risk for recurrent cardiovascular events.

## Funding support and author disclosures

The design and conduct of the study, interpretation of the results, and preparation of the manuscript were supported through a research grant from 10.13039/100002429Amgen, Inc. Dr Colantonio has received research support from Amgen Inc. Dr Jones, Dr Dhalwani, Ms Shannon, and Dr Kalich are employees and stockholders of Amgen Inc. Dr Muntner has received research support and consulting fees from Amgen Inc. Dr Rosenson has received research grants to his institution from Amgen, Arrowhead, Eli Lilly, NIH, Novartis and Regeneron; consulting fees from Amgen, CRISPER Therapeutics Eli Lilly, Lipigon, Novartis, Precision Biosciences, Regeneron, UltraGenyx, and Verve; non-promotional honoraria from Kowa; royalties from Wolters Kluwer (UpToDate); stock holding in MediMergent, LLC; and patent applications on: Methods and systems for biocellular marker detection and diagnosis using a microfluidic profiling device (EFS ID: 32278349, Application No. PCT/US2019/026364, provisional); compositions and methods relating to the identification and treatment of immunothrombotic conditions (New International Application No. PCT/US2021/63104926), and Quantification of Lp(a) vs non-Lp(a) apoB concentration: development of a novel validated equation. (Application No. PCT/US2021/63248837). Dr Bittner has received research grants to her institution from Sanofi and Regeneron (ODYSSEY OUTCOMES trial, Steering Committee member), Esperion (CLEAR OUTCOMES trial, National Coordinator), Dalcor (DalGene trial, National Coordinator), Astra Zeneca (STRENGTH trial, National Coordinator), Novartis (ORION trial, Site PI), Amgen (Industry Collaboration between the UAB School of Public Health and Amgen, co-Investigator); and has received payments as Senior Guest Editor for Circulation (American Heart Association, ongoing), Editor in Chief of the American College of Cardiology Self-Assessment Program (ACCSAP, American College of Cardiology, ongoing), Advisory Board member (Pfizer, concluded at the end of 2021), and Data Safety Monitoring Board member (Verve Therapeutics, ongoing). All other authors have reported that they have no relationships relevant to the contents of this paper to disclose.

PERSPECTIVES **COMPETENCY IN PATIENT CARE AND PROCEDURAL SKILLS:** LDL-C testing following MI hospital discharge among Medicare beneficiaries was low. There were disparities in LDL-C testing following MI hospital discharge by race/ethnicity (lower in Black vs White beneficiaries), geographic region of residence (highest in the Middle Atlantic and lowest in the West North Central U.S. regions), and type of Medicare program (lower in beneficiaries without vs with pharmacy benefits).

**TRANSLATIONAL OUTLOOK:** Increasing the use of LDL-C testing among Medicare beneficiaries following an MI may lead to guideline-recommended lipid-lowering therapy and a reduction of the risk for recurrent cardiovascular events.
