# Supplementary material for: Low-Density Lipoprotein Cholesterol Testing Following Myocardial Infarction Hospitalization Among Medicare Beneficiaries
Source: JACC Adv. 2023 Dec 8;3(1):100753. doi: 10.1016/j.jacadv.2023.100753 (PMC11198160; doi:10.1016/j.jacadv.2023.100753)
Supplement: Supplemental Tables 1-8 and Supplemental Figures 1-7 [file mmc1.docx]

SUPPLEMENTAL APPENDIX

**Low-Density Lipoprotein Cholesterol Testing Following Myocardial Infarction Hospitalization Among Medicare Beneficiaries**

Lisandro D. Colantonio, MD PhD, Zhixin Wang, MS, Jenna Jones, PhD,
Nafeesa Dhalwani, PhD, Erin D. Shannon, MPH, Cici Liu, MS, Bethany A. Kalich, PharmD, Paul Muntner, PhD, Robert S. Rosenson, MD, Vera Bittner, MD, MSPH

## Supplemental Table 1.

Current procedural terminology codes used to define a lipid test in sensitivity analyses

| **CPT code** | **Description** |
| --- | --- |
| **Codes used in the main analysis and in the sensitivity analysis** | |
| 80061 | Lipid panel including total cholesterol, high-density lipoprotein cholesterol and triglycerides. |
| 83721 | Low-density lipoprotein cholesterol, direct measurement. |
| **Codes used in the sensitivity analysis only** | |
| 82465 | Total cholesterol. |
| 83718 | High-density lipoprotein cholesterol. |
| 84478 | Triglycerides. |
| 83695 | Lipoprotein(a). |
| 83700 | Lipoprotein, blood; electrophoretic separation and quantitation |
| 83704 | Lipoprotein, blood; quantitation of lipoprotein particle numbers and lipoprotein particle subclasses (e.g., by nuclear magnetic resonance spectroscopy). |
| 82172 | Apolipoprotein B |
| CPT: current procedural terminology. | |

## Supplemental Table 2.

Definition of patient characteristics

| **Patient characteristic** | **Definition** |
| --- | --- |
| Calendar year of the MI hospital discharge | Defined based on the discharge date in the inpatient claim. |
| Age | Calculated on each patient’s MI discharge date using date of birth. |
| Race/ethnicity | Defined using beneficiary enrollment data. |
| Sex | Defined using beneficiary enrollment data. |
| Place of residence | Using zip codes in the beneficiary enrollment data, we defined county and State of residence. Using State of residence data, geographic region of residence was defined as follows:   1. New England: Connecticut, Maine, Vermont, Rhode Island, New Hampshire, and Massachusetts. 2. Middle Atlantic: New Jersey, New York, and Pennsylvania. 3. West South Central: Texas, Louisiana, Oklahoma, and Arkansas. 4. East South Central: Tennessee, Mississippi, Alabama, and Kentucky 5. South Atlantic: West Virginia, Georgia, Virginia, South and North Carolina, Maryland, Washington DC, Delaware, and Florida. 6. West North Central: South and North Dakota, Iowa, Missouri, Kansas, Nebraska, and Minnesota. 7. East North Central: Wisconsin, Illinois, Michigan, Ohio, and Indiana. 8. Mountain: Wyoming, Montana, Idaho, New Mexico, Utah, Arizona, Nevada, and Colorado. 9. Pacific: Oregon, Hawaii, Alaska, California, and Washington   For more details, please visit: <https://www.census.gov/geographies/reference-maps/2010/geo/2010-census-regions-and-divisions-of-the-united-states.html> |
| Dual Medicare-Medicaid eligibility/Low-income subsidy for prescription medications | Dual eligibility was defined by a continuous dual Medicare-Medicaid eligible status code of 01-08 during the 365 days prior to each beneficiary’s MI admission date. Low-income subsidy for prescription medications was defined by a continuous medication cost sharing group code of 01-08 during the 365 days prior to each beneficiary’s MI admission date. |
| Diabetes^1-3^ | Any of the following using all available claims prior to each patient’s MI discharge date, inclusive:  Algorithm based on ICD-9 codes:   1. At least 1 inpatient claim with a discharge ICD-9 diagnosis (any position) of 250.xx, 357.2, 362.0x, or 366.41. 2. At least 2 carrier claims, carrier line or outpatient claims with ICD-9 diagnoses (any position) of 250.xx, 357.2, 362.0x, or 366.41, linked by CLAIM_ID to an ambulatory physician evaluation and management claim, with the 2 claims occurring at least 7 days apart. 3. At least 1 pharmacy claim for an oral antidiabetic drug fill or insulin.   Algorithm based on ICD-10 codes:   1. At least 1 inpatient claim with a discharge ICD-10 diagnosis (any position) of 'E0836', 'E0842', 'E0936', 'E0942', 'E1010', 'E1011', 'E1029', 'E10311', 'E10319', 'E1036', 'E1039', 'E1040', 'E1042', 'E1051', 'E10618', 'E10620', 'E10621', 'E10622', 'E10628', 'E10630', 'E10638', 'E10641', 'E10649', 'E1065', 'E1069', 'E108', 'E109', 'E1100', 'E1101', 'E1129', 'E11311', 'E11319', 'E11329', 'E11339', 'E11349', 'E11359', 'E1136', 'E1139', 'E1140', 'E1142', 'E1151', 'E11618', 'E11620', 'E11621', 'E11622', 'E11628', 'E11630', 'E11638', 'E11641', 'E11649', 'E1165', 'E1169', 'E118', 'E119', 'E1310', 'E1336', 'E1342', 'E1037X1', 'E1037X2', 'E1037X3', 'E1037X9', 'E1110', 'E1111', 'E113291', 'E113292', 'E113293', 'E113299', 'E113391', 'E113392', 'E113393', 'E113399', 'E113491', 'E113492', 'E113493', 'E113499', 'E113591', 'E113592', 'E113593', 'E113599', 'E1137X2'. 2. At least 2 carrier claims, carrier line or outpatient claims with ICD-10 diagnoses (any position) of 'E0836', 'E0842', 'E0936', 'E0942', 'E1010', 'E1011', 'E1029', 'E10311', 'E10319', 'E1036', 'E1039', 'E1040', 'E1042', 'E1051', 'E10618', 'E10620', 'E10621', 'E10622', 'E10628', 'E10630', 'E10638', 'E10641', 'E10649', 'E1065', 'E1069', 'E108', 'E109', 'E1100', 'E1101', 'E1129', 'E11311', 'E11319', 'E11329', 'E11339', 'E11349', 'E11359', 'E1136', 'E1139', 'E1140', 'E1142', 'E1151', 'E11618', 'E11620', 'E11621', 'E11622', 'E11628', 'E11630', 'E11638', 'E11641', 'E11649', 'E1165', 'E1169', 'E118', 'E119', 'E1310', 'E1336', 'E1342', 'E1037X1', ‘E1037X2', 'E1037X3', 'E1037X9', 'E1110', 'E1111', 'E113291', 'E113292', 'E113293', 'E113299', 'E113391', 'E113392', 'E113393', 'E113399', 'E113491', 'E113492', 'E113493', 'E113499', 'E113591', 'E113592', 'E113593', 'E113599', 'E1137X2', linked by CLAIM_ID to an ambulatory physician evaluation and management claim, with the 2 claims occurring at least 7 days apart. 3. At least 1 pharmacy claim for an oral antidiabetic drug fill or insulin. |
| Chronic kidney disease (CKD)^4,5^ | Any of the following using all available claims prior to each patient’s MI discharge date, inclusive:  Algorithm based on ICD-9 codes:   1. ≥1 inpatient claim with a discharge diagnosis code of chronic kidney disease (ICD-9 diagnosis code of 016.0x, 095.4, 189.0, 189.9, 223.0, 236.91, 250.4x, 271.4, 274.1x, 283.11, 403.x1, 403.x0, 404.x2, 404.x3, 404.x0, 404.x1, 440.1, 442.1, 447.3, 572.4, 580.xx–588.xx, 591, 642.1x, 646.2x, 753.12–753.17, 753.19, 753.2x, 794.4) in any discharge diagnosis position. 2. ≥1 physician evaluation and management visit with a diagnosis code of chronic kidney disease (ICD-9 diagnosis code of 016.0x, 095.4, 189.0, 189.9, 223.0, 236.91, 250.4x, 271.4, 274.1x, 283.11, 403.x1, 403.x0, 404.x2, 404.x3, 404.x0, 404.x1, 440.1, 442.1, 447.3, 572.4, 580.xx–588.xx, 591, 642.1x, 646.2x, 753.12–753.17, 753.19, 753.2x, 794.4) in any position. 3. For Medicare data, if the flag ESRD_IND in the Master beneficiary summary file is “Y”, then the patient will be categorized as having a history of CKD.   Algorithm based on ICD-10 codes:   1. ≥1 inpatient claim with a discharge diagnosis code of chronic kidney disease (ICD-10 diagnosis code of ‘A1811', 'A5275', 'C649', 'C689', 'D3000', 'D4100', 'D4120', 'D593', 'E1021’, 'E1029', 'E1121’, 'E1129', 'E748', 'I120', 'I129', 'I130', 'I1310', 'I1311', 'I132', I701', 'I722', 'K767', 'M1030', 'N003', 'N008', 'N009', 'N013', 'N022', 'N032', 'N033', 'N035', 'N038', 'N039', 'N040', 'N043', 'N044', 'N048', 'N049', 'N052', 'N055', 'N058', 'N059', 'N08', 'N1330', 'N170', 'N171', 'N172', 'N178', 'N179', 'N181', 'N182', 'N183', 'N184', 'N185', 'N186', 'N189', 'N19', 'N250', 'N251', 'N2581', 'N2589', 'N259', 'N269','Q6102', 'Q6119', 'Q612', 'Q613', 'Q614', 'Q615', 'Q618', 'Q6210', 'Q6211', 'Q6212', 'Q6231', 'Q6239', 'R944') in any discharge diagnosis position. 2. ≥1 physician evaluation and management visit with a diagnosis code of chronic kidney disease (ICD-10 diagnosis code of ‘A1811', 'A5275', 'C649', 'C689', 'D3000', 'D4100', 'D4120', 'D593', 'E1021’, 'E1029', 'E1121’, 'E1129', 'E748', 'I120', 'I129', 'I130', 'I1310', 'I1311', 'I132', I701', 'I722', 'K767', 'M1030', 'N003', 'N008', 'N009', 'N013', 'N022', 'N032', 'N033', 'N035', 'N038', 'N039', 'N040', 'N043', 'N044', 'N048', 'N049', 'N052', 'N055', 'N058', 'N059', 'N08', 'N1330', 'N170', 'N171', 'N172', 'N178', 'N179', 'N181', 'N182', 'N183', 'N184', 'N185', 'N186', 'N189', 'N19', 'N250', 'N251', 'N2581', 'N2589', 'N259', 'N269','Q6102', 'Q6119', 'Q612', 'Q613', 'Q614', 'Q615', 'Q618', 'Q6210', 'Q6211', 'Q6212', 'Q6231', 'Q6239', 'R944') in any position.   (c) For Medicare data, if the flag ESRD_IND in the Master beneficiary summary file is “Y”, the patient will be categorized as having a history of CKD. |
| Heart failure^6^ | Any of the following using all available claims prior to each patient’s MI discharge date, inclusive:  Algorithm based on ICD-9 codes:  a) ≥ 1 inpatient claim with ICD-9 diagnoses (any position) of 402.01, 402.11, 402.91, 404.01, 404.03, 404.11, 404.13, 404.91, 404.93, 428.X.  b) ≥ 2 outpatient or carrier claims on separate days with ICD-9 diagnoses (any position) of 402.01, 402.11, 402.91, 404.01, 404.03, 404.11, 404.13, 404.91, 404.93, 428.X, linked (by CLAIM_ID in Medicare) to an ambulatory physician evaluation and management claim.  Algorithm based on ICD-10 codes:  a) ≥ 1 inpatient claim with ICD-10 diagnoses (any position) of 'I110', 'I130', 'I132', 'I501', 'I5020', 'I5021', 'I5022', 'I5023', 'I5030', 'I5031', 'I5032', 'I5033', 'I5040', 'I5041', 'I5042', 'I5043', 'I509', 'I50810', 'I50814', 'I50811', 'I50812', 'I50813', 'I5082', 'I5083', 'I5084', 'I5089'.  (b) ≥ 2 outpatient or carrier claims on separated days with ICD-10 diagnoses (any position) of 'I110', 'I130', 'I132', 'I501', 'I5020', 'I5021', 'I5022', 'I5023', 'I5030', 'I5031', 'I5032', 'I5033', 'I5040', 'I5041', 'I5042', 'I5043', 'I509', 'I50810', 'I50814', 'I50811', 'I50812', 'I50813', 'I5082', 'I5083', 'I5084', 'I5089', linked (by CLAIM_ID in Medicare) to an ambulatory physician evaluation and management claim. |
| History of stroke^7^ | Any of the following using all available claims prior to each patient’s MI discharge date, inclusive:   1. ≥1 overnight inpatient claim with a discharge diagnosis code for stroke (ICD-9 diagnosis codes 430.xx, 431.xx, 433.x1, 434.x1 or 436.x, or an ICD-10 diagnosis code of 'I60.xx', 'I61.xx', 'I63.xx', ‘I67.89’, ‘I67850’, ‘I67858’) in any discharge diagnosis position. 2. ≥2 EM-linked outpatient claims on separate days with an ICD-9 diagnosis code of 430.xx, 431.xx, 433.x1, 434.x1 or 436.x, or an ICD-10 diagnosis code of 'I60.xx', 'I61.xx', 'I63.xx', ‘I67.89’, ‘I67850’, ‘I67858’) in any discharge diagnosis position. |
| Lower extremity artery disease event (LEAD)^8^ | Any of the following using all available claims prior to each patient’s MI discharge date, inclusive:   1. ≥1 hospitalization with a discharge diagnosis code of atherosclerosis or thrombosis of arteries of the extremities (ICD-9 diagnosis code of 440.2, 440.20, 440.21, 440.22, 440.23, 440.24, 440.29, 440.3, 440.30, 440.31, 440.32, 440.4, 443.9, or ICD-10 diagnosis code of I70.2, I70.20, I70.201, I70.202, I70.203, I70.208, I70.209, I70.21, I70.211, I70.212, I70.213, I70.218, I70.219, I70.22, I70.221, I70.222, I70.223, I70.228, I70.229, I70.23, I70.231, I70.232, I70.233, I70.234, I70.235, I70.238, I70.239, I70.24, I70.241, I70.242, I70.243, I70.244, I70.245, I70.248, I70.249, I70.25, I70.26, I70.261, I70.262, I70.263, I70.268, I70.269, I70.29, I70.291, I70.292, I70.293, I70.298, I70.299, I70.3, I70.30, I70.301, I70.302, I70.303, I70.308, I70.309, I70.31, I70.311, I70.312, I70.313, I70.318, I70.319, I70.32, I70.321, I70.322, I70.323, I70.328, I70.329, I70.33, I70.331, I70.332, I70.333, I70.334, I70.335, I70.338, I70.339, I70.34, I70.341, I70.342, I70.343, I70.344, I70.345, I70.348, I70.349, I70.35, I70.36, I70.361, I70.362, I70.363, I70.368, I70.369, I70.39, I70.391, I70.392, I70.393, I70.398, I70.399, I70.4, I70.40, I70.401, I70.402, I70.403, I70.408, I70.409, I70.41, I70.411, I70.412, I70.413, I70.418, I70.419, I70.42, I70.421, I70.422, I70.423, I70.428, I70.429, I70.43, I70.431, I70.432, I70.433, I70.434, I70.435, I70.438, I70.439, I70.44, I70.441, I70.442, I70.443, I70.444, I70.445, I70.448, I70.449, I70.45, I70.46, I70.461, I70.462, I70.463, I70.468, I70.469, I70.49, I70.491, I70.492, I70.493, I70.498, I70.499, I70.5, I70.50, I70.501, I70.502, I70.503, I70.508, I70.509, I70.51, I70.511, I70.512, I70.513, I70.518, I70.519, I70.52, I70.521, I70.522, I70.523, I70.528, I70.529, I70.53, I70.531, I70.532, I70.533, I70.534, I70.535, I70.538, I70.539, I70.54, I70.541, I70.542, I70.543, I70.544, I70.545, I70.548, I70.549, I70.55, I70.56, I70.561, I70.562, I70.563, I70.568, I70.569, I70.59, I70.591, I70.592, I70.593, I70.598, I70.599, I70.6, I70.60, I70.601, I70.602, I70.603, I70.608, I70.609, I70.61, I70.611, I70.612, I70.613, I70.618, I70.619, I70.62, I70.621, I70.622, I70.623, I70.628, I70.629, I70.63, I70.631, I70.632, I70.633, I70.634, I70.635, I70.638, I70.639, I70.64, I70.641, I70.642, I70.643, I70.644, I70.645, I70.648, I70.649, I70.65, I70.66, I70.661, I70.662, I70.663, I70.668, I70.669, I70.69, I70.691, I70.692, I70.693, I70.698, I70.699, I70.7, I70.70, I70.701, I70.702, I70.703, I70.708, I70.709, I70.71, I70.711, I70.712, I70.713, I70.718, I70.719, I70.72, I70.721, I70.722, I70.723, I70.728, I70.729, I70.73, I70.731, I70.732, I70.733, I70.734, I70.735, I70.738, I70.739, I70.74, I70.741, I70.742, I70.743, I70.744, I70.745, I70.748, I70.749, I70.75, I70.76, I70.761, I70.762, I70.763, I70.768, I70.769, I70.79, I70.791, I70.792, I70.793, I70.798, I70.799, I70.9, I70.92, I739) in any discharge diagnosis position. 2. ≥2 physician evaluation and management visits with a diagnosis code of atherosclerosis or thrombosis of arteries of the extremities (ICD-9 diagnosis code of 440.2, 440.20, 440.21, 440.22, 440.23, 440.24, 440.29, 440.3, 440.30, 440.31, 440.32, 440.4, 443.9, or ICD-10 diagnosis code of I70.2, I70.20, I70.201, I70.202, I70.203, I70.208, I70.209, I70.21, I70.211, I70.212, I70.213, I70.218, I70.219, I70.22, I70.221, I70.222, I70.223, I70.228, I70.229, I70.23, I70.231, I70.232, I70.233, I70.234, I70.235, I70.238, I70.239, I70.24, I70.241, I70.242, I70.243, I70.244, I70.245, I70.248, I70.249, I70.25, I70.26, I70.261, I70.262, I70.263, I70.268, I70.269, I70.29, I70.291, I70.292, I70.293, I70.298, I70.299, I70.3, I70.30, I70.301, I70.302, I70.303, I70.308, I70.309, I70.31, I70.311, I70.312, I70.313, I70.318, I70.319, I70.32, I70.321, I70.322, I70.323, I70.328, I70.329, I70.33, I70.331, I70.332, I70.333, I70.334, I70.335, I70.338, I70.339, I70.34, I70.341, I70.342, I70.343, I70.344, I70.345, I70.348, I70.349, I70.35, I70.36, I70.361, I70.362, I70.363, I70.368, I70.369, I70.39, I70.391, I70.392, I70.393, I70.398, I70.399, I70.4, I70.40, I70.401, I70.402, I70.403, I70.408, I70.409, I70.41, I70.411, I70.412, I70.413, I70.418, I70.419, I70.42, I70.421, I70.422, I70.423, I70.428, I70.429, I70.43, I70.431, I70.432, I70.433, I70.434, I70.435, I70.438, I70.439, I70.44, I70.441, I70.442, I70.443, I70.444, I70.445, I70.448, I70.449, I70.45, I70.46, I70.461, I70.462, I70.463, I70.468, I70.469, I70.49, I70.491, I70.492, I70.493, I70.498, I70.499, I70.5, I70.50, I70.501, I70.502, I70.503, I70.508, I70.509, I70.51, I70.511, I70.512, I70.513, I70.518, I70.519, I70.52, I70.521, I70.522, I70.523, I70.528, I70.529, I70.53, I70.531, I70.532, I70.533, I70.534, I70.535, I70.538, I70.539, I70.54, I70.541, I70.542, I70.543, I70.544, I70.545, I70.548, I70.549, I70.55, I70.56, I70.561, I70.562, I70.563, I70.568, I70.569, I70.59, I70.591, I70.592, I70.593, I70.598, I70.599, I70.6, I70.60, I70.601, I70.602, I70.603, I70.608, I70.609, I70.61, I70.611, I70.612, I70.613, I70.618, I70.619, I70.62, I70.621, I70.622, I70.623, I70.628, I70.629, I70.63, I70.631, I70.632, I70.633, I70.634, I70.635, I70.638, I70.639, I70.64, I70.641, I70.642, I70.643, I70.644, I70.645, I70.648, I70.649, I70.65, I70.66, I70.661, I70.662, I70.663, I70.668, I70.669, I70.69, I70.691, I70.692, I70.693, I70.698, I70.699, I70.7, I70.70, I70.701, I70.702, I70.703, I70.708, I70.709, I70.71, I70.711, I70.712, I70.713, I70.718, I70.719, I70.72, I70.721, I70.722, I70.723, I70.728, I70.729, I70.73, I70.731, I70.732, I70.733, I70.734, I70.735, I70.738, I70.739, I70.74, I70.741, I70.742, I70.743, I70.744, I70.745, I70.748, I70.749, I70.75, I70.76, I70.761, I70.762, I70.763, I70.768, I70.769, I70.79, I70.791, I70.792, I70.793, I70.798, I70.799, I70.9, I70.92, I739) in any discharge position on separate days. 3. ≥1 hospitalization or physician visit with a CPT code of 37205, 75962, 36902, 36905, 37246, or 37247. |
| Recent acute coronary syndrome^9^ | Overnight hospitalization with a discharge diagnosis code for myocardial infarction (ICD-9 codes 410.x0 or 410.x1 or ICD-10 codes I21.xx or I22.xx) or unstable angina (ICD-9 codes 411.1, 411.81, 411.89 or ICD-10 codes I20.0, I24.0, I24.8, I24.9, I25.110, I25.700, I25.710, I25.720, I25.730, I25.750, I25.760, I25.790) in any position in the 365 days prior to each patient’s MI admission date. |
| Coronary heart disease^9^ | Any of the following using all available claims prior to each patient’s MI admission date:  (a) At least 1 inpatient claim with an ICD-9 diagnosis code of 410.xx-414.xx, V45.81 or V45.82, or an ICD-10 diagnosis code of I21.xxx, I22.xxx, I25.10, I25.810, I25.811, I25.812, I25.3, I25.41, I25.42, Z95.1, Z9861, I200, I201, I208, I209, I240, I241, I248, I252, I255, I2582, I2583, I2584, I2589, I259, in any position.  (b) At least 1 outpatient physician evaluation and management claim with an ICD-9 diagnosis code of 410.xx-414.xx, V45.81 or V45.82, or an ICD-10 diagnosis code of I21.xxx, I22.xxx, I25.10, I25.810, I25.811, I25.812, I25.3, I25.41, I25.42, Z95.1, Z9861, I200, I201, I208, I209, I240, I241, I248, I252, I255, I2582, I2583, I2584, I2589, I259, in any position.  (c) At least 1 inpatient or outpatient claim with an ICD-9 procedure code of 00.66, 36.0, 36.01-36.19, 36.2, or an ICD-10 procedure code of '0210xxx', '0211xxx', '0212xxx', ‘0213xxx', '0270xxx', '0271xxx', '0272xxx', '0273xxx','02C0xxx', '02C1xxx', '02C2xxx', '02C3xxx', '3E07xxx', or a HCPCS code of 33510-33519, 33521-33523, 33530, 33533-33536, 92980-92982, 92984, 92995, 92996, 92920, 92921, 92924, 92925, 92928, 92929, 92933, 92934, 92937, 92938, 92941, 92943, 92944, 92973, C9600, C9601, C9602, C9603, C9604, C9605, C9606, C9607, C9608, G0290, G0291. |
| Coronary revascularization procedure during the MI hospitalization (includes coronary artery bypass grafting or percutaneous coronary intervention) | Any of following excluding those occurring during the MI hospitalization:   - 1. ≥1 inpatient claim with a discharge diagnosis code of coronary artery bypass grafting or percutaneous coronary intervention (ICD-9 diagnosis code of V45.81, V45.82, or ICD-10 code of Z951, Z9861) in any discharge diagnosis position.   2. ≥1 outpatient claims with a diagnosis code for coronary artery bypass grafting or percutaneous coronary intervention (ICD-9 diagnosis code of V45.81, V45.82, or ICD-10 code of Z951, Z9861).   3. ≥1 inpatient or outpatient claim with an ICD-9 procedure code of 00.66, 36.0, 36.01-36.19, 36.2, or an ICD-10 procedure code of '0210xxx', '0211xxx', '0212xxx', ‘0213xxx', '0270xxx', '0271xxx', '0272xxx', '0273xxx', '02C0xxx', '02C1xxx', '02C2xxx', '02C3xxx', '3E07xxx', or a HCPCS code of 33510-33519, 33521-33523, 33530, 33533-33536, 92980-92982, 92984, 92995, 92996, 92920, 92921, 92924, 92925, 92928, 92929, 92933, 92934, 92937, 92938, 92941, 92943, 92944, 92973, C9600, C9601, C9602, C9603, C9604, C9605, C9606, C9607, C9608, G0290, G0291. |
| **Medication use defined using pharmacy claims in the 90 days prior to each patient’s MI hospital admission date and within 7 days after each patient’s MI hospital discharge date** | |
| Statin, intensity of therapy | Defined based on the highest intensity for a statin fill including all pharmacy claims for a statin within the 90 days prior to each patient’s MI hospital admission date, inclusive, and within the 7 days after each patient’s MI hospital discharge date, inclusive. The statin intensity was categorized as follows based on the 2018 American Heart Association/American College of Cardiology cholesterol guideline:   - None - Low/moderate intensity - High intensity |
| Statin, initiation | Defined by meeting the 2 following conditions:   1. Having no fills for a statin on the MI hospital admission date or within the 90 days prior to the MI hospital admission date, and 2. Having a fill for a statin on the MI hospital discharge date or within 7 days after each patient’s MI hospital discharge date. |
| Statin, up-titration | Defined by meeting the 3 following conditions:   1. Having a fill for a low- or moderate-intensity statin on the MI hospital admission date or within the 90 days prior to the MI hospital admission date, and 2. Having no fills for a high-intensity statin on the MI hospital admission date or within the 90 days prior to the MI hospital admission date, and 3. Having a fill for a high-intensity statin on the MI hospital discharge date or within 7 days after each patient’s MI hospital discharge date. |
| Ezetimibe, prevalent use | Defined by having a fill for ezetimibe on the MI hospital admission date or within the 90 days prior to the MI hospital admission date. |
| Ezetimibe, initiation | Defined by meeting the 2 following conditions:   1. Having no fills for ezetimibe on the MI hospital admission date or within the 90 days prior to the MI hospital admission date, and 2. Having a fill for ezetimibe on the MI hospital discharge date or within 7 days after each patient’s MI hospital discharge date. |
| Use of PCSK9mAb* | Defined by having a fill for a PCSK9mAb within the 90 days prior to each patient’s MI hospital admission date, inclusive, or within the 7 days after each patient’s MI hospital discharge date, inclusive. |
| **Patient characteristics defined using claims in the 30 days after each patient’s MI hospital discharge date†** | |
| Cardiologist care | Defined by an outpatient or carrier claim for an ambulatory physician evaluation and management visit with a specialty code of 06 (cardiology). |
| Primary care ambulatory visit | Defined by an outpatient or carrier claim for an ambulatory physician evaluation and management visit with a specialty code of 01 (general practice), 08 (family practice), or 11 (internal medicine). |
| Re-hospitalization | Any inpatient claim. |
| Recurrent MI | An inpatient claim for an overnight stay with an ICD-10 discharge diagnosis code of I21.xxx or I22.xxx in any discharge diagnosis position. |
| Coronary revascularization procedure | See codes above for “Coronary revascularization procedure during the MI hospitalization”. |
| Cardiac rehabilitation | Defined by a claim with CPT/HCPCS codes of 93797, 93798, G0422, or G0423. |
| CPT: Current Procedure Terminology; HCPSC: Healthcare Common Procedure Coding System; ICD-9: international classification of diseases, ninth revision; ICD-10: international classification of diseases, tenth revision; LDL-C: low-density lipoprotein cholesterol; MI: myocardial infarction; PCSK9mAb: proprotein convertase subtilisin/kexin type 9 monoclonal antibody.  * PCSK9mAbs were referred as proprotein convertase subtilisin/kexin type 9 inhibitors in the 2018 American Heart Association (AHA)/American College of Cardiology (ACC)/Multi-society guideline on the management of blood cholesterol.^10^    **†** For patients with an LDL-C test within 30 days after their MI hospital discharge date, patient characteristics in the 30 days after their MI hospital discharge date were considered to be present only if these preceded or may have led to the LDL-C test. Specifically, we considered cardiologist care and primary care ambulatory visits to have occurred only if patients did not have an LDL-C test between their MI hospital discharge date and 3 days before their earliest cardiologist or primary care physician visits, respectively. We included outpatient cardiologist and primary care physician visits up to 3 days after an LDL-C test as this may have been ordered in advance of the visit. We considered re-hospitalization, recurrent MI, coronary revascularization procedure, and cardiac rehabilitation to have occurred only if patients did not have an LDL-C test between their MI hospital discharge date and the date of their earliest re-hospitalization, recurrent MI, coronary revascularization procedure, or cardiac rehabilitation session, respectively. | |

## Supplemental Table 3.

Characteristic of patients in the fee-for-service with pharmacy benefits cohort with and without an LDL-C test within 30 days following MI hospital discharge

|  | LDL-C test within 30 days following MI hospital discharge | |
| --- | --- | --- |
| Patient characteristics | No  n=475,541 | Yes  n=57,226 |
| Calendar year of the MI hospital discharge, n (%) |  |  |
| 2016 | 85,751 (18.0) | 10,593 (18.5) |
| 2017 | 93,342 (19.6) | 11,288 (19.7) |
| 2018 | 98,315 (20.7) | 12,169 (21.3) |
| 2019 | 104,035 (21.9) | 12,474 (21.8) |
| 2020 | 94,098 (19.8) | 10,702 (18.7) |
| Age, n (%) |  |  |
| 66 to 75 years | 229,407 (48.2) | 29,334 (51.3) |
| ≥76 years | 246,134 (51.8) | 27,892 (48.7) |
| Male, n (%) | 249,922 (52.6) | 31,921 (55.8) |
| Race/ethnicity, n (%) |  |  |
| Non-Hispanic white | 401,740 (84.5) | 47,751 (83.4) |
| Non-Hispanic Black | 40,112 (8.4) | 4,490 (7.8) |
| Asian | 9,203 (1.9) | 1,363 (2.4) |
| Hispanic | 9,056 (1.9) | 1,429 (2.5) |
| Other | 15,430 (3.2) | 2,193 (3.8) |
| Geographic region of residence, n (%) |  |  |
| New England | 31,489 (6.6) | 3,496 (6.1) |
| West South Central | 52,460 (11.0) | 6,329 (11.1) |
| Mountain | 24,426 (5.1) | 2,647 (4.6) |
| East South Central | 35,472 (7.5) | 4,262 (7.4) |
| Middle Atlantic | 62,224 (13.1) | 10,294 (18.0) |
| South Atlantic | 97,371 (20.5) | 12,431 (21.7) |
| West North Central | 34,809 (7.3) | 2,978 (5.2) |
| East North Central | 83,112 (17.5) | 7,660 (13.4) |
| Pacific | 54,178 (11.4) | 7,129 (12.5) |
| Dual eligibility/Low-income subsidy for medications, n (%) | 128,985 (27.1) | 14,876 (26.0) |
| Diabetes, n (%) | 223,767 (47.1) | 30,825 (53.9) |
| Chronic kidney disease, n (%) | 272,078 (57.2) | 33,400 (58.4) |
| Heart failure, n (%) | 251,216 (52.8) | 29,499 (51.5) |
| History of stroke, n (%) | 37,125 (7.8) | 4,025 (7.0) |
| History of lower extremity artery disease, n (%) | 103,574 (21.8) | 12,748 (22.3) |
| Recent ACS, n (%) | 68,354 (14.4) | 8,050 (14.1) |
| History of CHD, n (%) | 285,839 (60.1) | 35,140 (61.4) |
| Coronary revascularization during the MI hospitalization, n (%) | 253,251 (53.3) | 34,822 (60.8) |
| **Medication use*** | | |
| Statin use |  |  |
| Intensity, n (%) |  |  |
| None | 119,828 (25.2) | 11,910 (20.8) |
| Low/Moderate-intensity therapy | 139,009 (29.2) | 16,764 (29.3) |
| High-intensity therapy | 216,704 (45.6) | 28,552 (49.9) |
| Initiation, n (%) | 110,826 (23.3) | 14,040 (24.5) |
| Up-titration, n (%) | 33,841 (7.1) | 4,985 (8.7) |
| Ezetimibe use, n (%) |  |  |
| Prevalent use | 13,234 (2.8) | 2,172 (3.8) |
| Initiation | 2,845 (0.6) | 497 (0.9) |
| PCSK9mAb† use, n (%) | 1,621 (0.3) | 359 (0.6) |
| **Patient characteristics defined using claims in the 30 days after each patient’s MI hospital discharge date‡** | | |
| Cardiologist outpatient visits, n (%) | 173,777 (36.5) | 20,295 (35.5) |
| Primary care physician outpatient visits, n (%) | 225,847 (47.5) | 27,436 (47.9) |
| Re-hospitalization, n (%) | 73,901 (15.5) | 3,691 (6.4) |
| Recurrent MI, n (%) | 23,804 (5.0) | 1,423 (2.5) |
| Coronary revascularization procedure, n (%) | 102,451 (21.5) | 11,611 (20.3) |
| Cardiac rehabilitation, n (%) | 38,612 (8.1) | 2,347 (4.1) |
| ACS: acute coronary syndrome; CHD: coronary heart disease; LDL-C: low-density lipoprotein cholesterol; MI: myocardial infarction; PCSK9mAb: proprotein convertase subtilisin/kexin type 9 monoclonal antibody.  The definition of LDL-C test includes an outpatient claim with a current procedural terminology code of 83721 or 80061.  * Medication use was defined using pharmacy claims in the 90 days prior to each patient’s MI hospital admission date through 7 days after each patient’s MI hospital discharge date. Definitions of medication use are provided in **Supplemental Table 2**.  † PCSK9mAbs were referred as proprotein convertase subtilisin/kexin type 9 inhibitors in the 2018 American Heart Association (AHA)/American College of Cardiology (ACC)/Multi-society guideline on the management of blood cholesterol.^10^  ‡ For patients with an LDL-C test within 30 days after their MI hospital discharge date, patient characteristics in the 30 days after their MI hospital discharge date were considered to be present only if these preceded or may have led to the LDL-C test. Specifically, we considered cardiologist care and primary care ambulatory visits to have occurred only if patients did not have an LDL-C test between their MI hospital discharge date and 3 days before their earliest cardiologist or primary care physician visits, respectively. We included outpatient cardiologist and primary care physician visits up to 3 days after an LDL-C test as this may have been ordered in advance of the visit. We considered re-hospitalization, recurrent MI, coronary revascularization procedure, and cardiac rehabilitation to have occurred only if patients did not have an LDL-C test between their MI hospital discharge date and the date of their earliest re-hospitalization, recurrent MI, coronary revascularization procedure, or cardiac rehabilitation session, respectively. | | |

## Supplemental Table 4.

Characteristic of patients in the fee-for-service with pharmacy benefits cohort with and without an LDL-C test within 365 days following MI hospital discharge

|  | LDL-C test within 365 days following MI hospital discharge | |
| --- | --- | --- |
| Patient characteristics | No  n=208,028 | Yes  n=324,739 |
| Calendar year of the MI hospital discharge, n (%) |  |  |
| 2016 | 32,284 (15.5) | 64,060 (19.7) |
| 2017 | 35,759 (17.2) | 68,871 (21.2) |
| 2018 | 38,116 (18.3) | 72,368 (22.3) |
| 2019 | 42,421 (20.4) | 74,088 (22.8) |
| 2020 | 59,448 (28.6) | 45,352 (14.0) |
| Age, n (%) |  |  |
| 66 to 75 years | 91,227 (43.9) | 167,514 (51.6) |
| ≥76 years | 116,801 (56.1) | 157,225 (48.4) |
| Male, n (%) | 105,098 (50.5) | 176,745 (54.4) |
| Race/ethnicity, n (%) |  |  |
| Non-Hispanic white | 172,251 (82.8) | 277,240 (85.4) |
| Non-Hispanic Black | 20,945 (10.1) | 23,657 (7.3) |
| Asian | 4,117 (2.0) | 6,449 (2.0) |
| Hispanic | 3,956 (1.9) | 6,529 (2.0) |
| Other | 6,759 (3.2) | 10,864 (3.3) |
| Geographic region of residence |  |  |
| New England | 14,563 (7.0) | 20,422 (6.3) |
| West South Central | 22,970 (11.0) | 35,819 (11.0) |
| Mountain | 11,595 (5.6) | 15,478 (4.8) |
| East South Central | 15,001 (7.2) | 24,733 (7.6) |
| Middle Atlantic | 24,144 (11.6) | 48,374 (14.9) |
| South Atlantic | 38,740 (18.6) | 71,062 (21.9) |
| West North Central | 16,894 (8.1) | 20,893 (6.4) |
| East North Central | 38,803 (18.7) | 51,969 (16.0) |
| Pacific | 25,318 (12.2) | 35,989 (11.1) |
| Dual eligibility/Low-income subsidy for medications, n (%) | 65,678 (31.6) | 78,183 (24.1) |
| Diabetes, n (%) | 92,663 (44.5) | 161,929 (49.9) |
| Chronic kidney disease, n (%) | 130,244 (62.6) | 175,234 (54.0) |
| Heart failure, n (%) | 124,123 (59.7) | 156,592 (48.2) |
| History of stroke, n (%) | 18,156 (8.7) | 22,994 (7.1) |
| History of lower extremity artery disease, n (%) | 49,319 (23.7) | 67,003 (20.6) |
| Recent ACS, n (%) | 34,241 (16.5) | 42,163 (13.0) |
| History of CHD, n (%) | 128,276 (61.7) | 192,703 (59.3) |
| Coronary revascularization during the MI hospitalization, n (%) | 91,089 (43.8) | 196,984 (60.7) |
| **Medication use*** | | |
| Statin use |  |  |
| Intensity, n (%) |  |  |
| None | 63,971 (30.8) | 67,767 (20.9) |
| Low/Moderate-intensity therapy | 58,476 (28.1) | 97,297 (30.0) |
| High-intensity therapy | 85,581 (41.1) | 159,675 (49.2) |
| Initiation, n (%) | 43,312 (20.8) | 81,554 (25.1) |
| Up-titration, n (%) | 11,164 (5.4) | 27,662 (8.5) |
| Ezetimibe use, n (%) |  |  |
| Prevalent use | 4,545 (2.2) | 10,861 (3.3) |
| Initiation | 894 (0.4) | 2,448 (0.8) |
| PCSK9mAb† use, n (%) | 468 (0.2) | 1,512 (0.5) |
| **Patient characteristics defined using claims in the 30 days after each patient’s MI hospital discharge date‡** | | |
| Cardiologist outpatient visits, n (%) | 63,725 (30.6) | 130,347 (40.1) |
| Primary care physician outpatient visits, n (%) | 95,097 (45.7) | 158,186 (48.7) |
| Re-hospitalization, n (%) | 39,034 (18.8) | 38,558 (11.9) |
| Recurrent MI, n (%) | 11,829 (5.7) | 13,398 (4.1) |
| Coronary revascularization procedure, n (%) | 37,390 (18.0) | 76,672 (23.6) |
| Cardiac rehabilitation, n (%) | 10,076 (4.8) | 30,883 (9.5) |
| ACS: acute coronary syndrome; CHD: coronary heart disease; LDL-C: low-density lipoprotein cholesterol; MI: myocardial infarction; PCSK9mAb: proprotein convertase subtilisin/kexin type 9 monoclonal antibody.  The definition of LDL-C test includes an outpatient claim with a current procedural terminology code of 83721 or 80061.  * Medication use was defined using pharmacy claims in the 90 days prior to each patient’s MI hospital admission date through 7 days after each patient’s MI hospital discharge date. Definitions of medication use are provided in **Supplemental Table 2**.  † PCSK9mAbs were referred as proprotein convertase subtilisin/kexin type 9 inhibitors in the 2018 American Heart Association (AHA)/American College of Cardiology (ACC)/Multi-society guideline on the management of blood cholesterol.^10^  ‡ For patients with an LDL-C test within 30 days after their MI hospital discharge date, patient characteristics in the 30 days after their MI hospital discharge date were considered to be present only if these preceded or may have led to the LDL-C test. Specifically, we considered cardiologist care and primary care ambulatory visits to have occurred only if patients did not have an LDL-C test between their MI hospital discharge date and 3 days before their earliest cardiologist or primary care physician visits, respectively. We included outpatient cardiologist and primary care physician visits up to 3 days after an LDL-C test as this may have been ordered in advance of the visit. We considered re-hospitalization, recurrent MI, coronary revascularization procedure, and cardiac rehabilitation to have occurred only if patients did not have an LDL-C test between their MI hospital discharge date and the date of their earliest re-hospitalization, recurrent MI, coronary revascularization procedure, or cardiac rehabilitation session, respectively. | | |

## Supplemental Table 5.

Cumulative incidence of an LDL-C test among patients in the fee-for-service with pharmacy benefits cohort by state

|  | Cumulative incidence, % | | | |
| --- | --- | --- | --- | --- |
| State | 30 days | 90 days | 365 days | |
| AK | 5.8 | 17.1 | 45.2 | |
| AL | 11.0 | 30.6 | 68.1 | |
| AZ | 12.7 | 32.5 | 67.2 | |
| AR | 9.7 | 26.8 | 64.4 | |
| CA | 13.0 | 33.2 | 66.1 | |
| CO | 7.0 | 26.3 | 61.8 | |
| CT | 11.0 | 29.8 | 64.4 | |
| DE | 9.7 | 29.9 | 68.7 | |
| DC | 9.8 | 25.2 | 54.0 | |
| FL | 14.0 | 38.4 | 74.7 | |
| GA | 11.2 | 31.7 | 68.6 | |
| HI | 19.3 | 42.6 | 74.4 | |
| ID | 6.6 | 20.2 | 53.2 | |
| IL | 8.6 | 26.5 | 64.3 | |
| IN | 7.0 | 23.7 | 60.3 | |
| IA | 6.7 | 21.3 | 60.4 | |
| KS | 7.2 | 21.9 | 58.2 | |
| KY | 10.2 | 28.9 | 64.3 | |
| LA | 10.4 | 29.1 | 64.1 | |
| ME | 6.4 | 22.9 | 57.1 | |
| MD | 10.8 | 30.4 | 65.6 | |
| MA | 11.0 | 30.4 | 65.1 | |
| MI | 9.2 | 25.7 | 61.1 | |
| MN | 6.9 | 21.7 | 56.0 | |
| MS | 9.7 | 26.9 | 64.1 | |
| MO | 9.4 | 26.1 | 62.5 | |
| MT | 5.6 | 18.4 | 52.7 | |
| NE | 8.1 | 23.6 | 60.8 | |
| NV | 12.1 | 33.3 | 67.0 | |
| NH | 8.8 | 29.0 | 64.0 | |
| NJ | 14.5 | 38.5 | 74.0 | |
| NM | 8.6 | 23.7 | 55.1 | |
| NY | 17.5 | 40.4 | 72.5 | |
| NC | 9.4 | 29.4 | 67.7 | |
| ND | 7.6 | 21.2 | 61.1 | |
| OH | 8.6 | 25.4 | 62.4 | |
| OK | 8.1 | 22.6 | 56.7 | |
| OR | 7.2 | 21.8 | 54.3 | |
| PA | 9.5 | 29.6 | 67.1 | |
| RI | 12.8 | 32.3 | 68.8 | |
| SC | 9.9 | 30.3 | 69.4 | |
| SD | 8.1 | 27.6 | 64.0 | |
| TN | 11.4 | 31.7 | 68.5 | |
| TX | 11.8 | 32.2 | 68.8 | |
| UT | 8.6 | 25.9 | 60.9 | |
| VT | 6.5 | 19.6 | 52.7 | |
| VA | 9.7 | 28.8 | 67.4 | |
| WA | 7.9 | 22.7 | 56.2 | |
| WV | 9.7 | 26.8 | 61.9 | |
| WI | 7.4 | 25.0 | 62.6 | |
| WY | 7.8 | 23.6 | 52.0 | |
| The definition of LDL-C test includes an outpatient claim with a current procedural terminology code of 83721 or 80061. | | | |  |

## Supplemental Table 6.

Patient characteristic associated with having an LDL-C test within 30 days and 365 days following hospital discharge for MI in the fee-for-service with pharmacy benefits cohort

| Patient characteristics | Ratio of predicted cumulative incidence at 30 days following MI hospital discharge (95% CI) | Ratio of predicted cumulative incidence at 365 days following MI hospital discharge (95% CI) |
| --- | --- | --- |
| Calendar year of the MI hospital discharge |  |  |
| 2016 | 1 (ref) | 1 (ref) |
| 2017 | 0.98 (0.96, 1.01) | 1.00 (0.99, 1.01) |
| 2018 | 1.01 (0.98, 1.03) | 1.00 (1.00, 1.01) |
| 2019 | 0.98 (0.95, 1.00) | 0.99 (0.98, 0.99) |
| 2020 | 0.93 (0.90, 0.95) | 0.98 (0.96, 0.99) |
| Age |  |  |
| 66 to 75 years | 1 (ref) | 1 (ref) |
| ≥76 years | 0.92 (0.90, 0.93) | 0.94 (0.91, 0.97) |
| Female | 1 (ref) | 1 (ref) |
| Male | 1.05 (1.03, 1.07) | 1.03 (1.01, 1.06) |
| Race/ethnicity |  |  |
| Non-Hispanic white | 1 (ref) | 1 (ref) |
| Non-Hispanic Black | 0.98 (0.96, 1.01) | 0.96 (0.91, 1.00) |
| Asian | 1.17 (1.11, 1.23) | 1.01 (0.94, 1.09) |
| Hispanic | 1.28 (1.22, 1.35) | 1.04 (0.96, 1.12) |
| Other | 1.11 (1.07, 1.16) | 0.99 (0.91, 1.08) |
| Geographic region of residence |  |  |
| New England | 1 (ref) | 1 (ref) |
| West South Central | 1.05 (1.01, 1.10) | 1.05 (0.99, 1.11) |
| Mountain | 0.96 (0.91, 1.01) | 0.96 (0.89, 1.03) |
| East South Central | 1.05 (1.00, 1.09) | 1.05 (0.99, 1.12) |
| Middle Atlantic | 1.38 (1.34, 1.44) | 1.08 (1.02, 1.15) |
| South Atlantic | 1.10 (1.06, 1.14) | 1.08 (1.02, 1.14) |
| West North Central | 0.84 (0.80, 0.87) | 0.93 (0.86, 1.00) |
| East North Central | 0.85 (0.82, 0.88) | 0.95 (0.89, 1.01) |
| Pacific | 1.12 (1.08, 1.17) | 1.03 (0.98, 1.09) |
| Dual eligibility/Low-income subsidy for medications | 0.89 (0.87, 0.90) | 0.94 (0.91, 0.97) |
| Diabetes | 1.24 (1.22, 1.26) | 1.07 (1.04, 1.10) |
| Chronic kidney disease | 1.06 (1.04, 1.08) | 0.93 (0.90, 0.95) |
| Heart failure | 0.98 (0.96, 0.99) | 0.88 (0.86, 0.90) |
| History of stroke | 0.90 (0.88, 0.93) | 0.97 (0.93, 1.02) |
| History of lower extremity artery disease | 1.00 (0.98, 1.02) | 0.99 (0.96, 1.02) |
| Recent ACS | 1.00 (0.98, 1.02) | 0.93 (0.89, 0.98) |
| History of CHD | 1.00 (0.98, 1.02) | 1.01 (0.98, 1.04) |
| Coronary revascularization during the MI hospitalization | 1.32 (1.30, 1.34) | 1.10 (1.07, 1.14) |
| **Medication use*** | |  |
| Statin use |  |  |
| Intensity |  |  |
| None | 1 (ref) | 1 (ref) |
| Low/moderate-intensity therapy | 1.14 (1.12, 1.17) | 1.14 (1.10, 1.19) |
| High-intensity therapy | 1.18 (1.16, 1.21) | 1.15 (1.11, 1.19) |
| Initiation (versus no initiation) | 1.03 (1.01, 1.05) | 0.95 (0.90, 1.00) |
| Up-titration (versus no up-titration) | 1.12 (1.09, 1.15) | 1.08 (1.03, 1.13) |
| Ezetimibe use |  |  |
| Prevalent use (versus no prevalent use) | 1.26 (1.21, 1.32) | 1.05 (0.92, 1.20) |
| Initiation (versus no initiation) | 1.39 (1.28, 1.50) | 0.97 (0.68, 1.38) |
| PCSK9mAb† use | 1.65 (1.48, 1.82) | 1.32 (1.29, 1.34) |
| **Patient characteristics defined using claims in the 30 days after each patient’s MI hospital discharge date‡** | |  |
| Cardiologist outpatient visits | 0.88 (0.86, 0.89) | 1.06 (1.03, 1.09) |
| Primary care physician outpatient visits | 1.04 (1.02, 1.05) | 1.02 (1.00, 1.05) |
| Re-hospitalization | 0.37 (0.36, 0.39) | 0.87 (0.84, 0.91) |
| Recurrent MI | 1.08 (1.05, 1.11) | 0.97 (0.91, 1.03) |
| Coronary revascularization procedures | 0.95 (0.93, 0.97) | 1.01 (0.96, 1.06) |
| Cardiac rehabilitation | 0.39 (0.37, 0.42) | 1.09 (1.01, 1.17) |
| ACS: acute coronary syndrome; CHD: coronary heart disease; CI: confidence interval; LDL-C: low-density lipoprotein cholesterol; MI: myocardial infarction; PCSK9mAb: proprotein convertase subtilisin/kexin type 9 monoclonal antibody.  The definition of LDL-C test includes an outpatient claim with a current procedural terminology code of 83721 or 80061.  Ratios include adjustment for all patient characteristics simultaneously.  * Medication use was defined using pharmacy claims in the 90 days prior to each patient’s MI hospital admission date through 7 days after each patient’s MI hospital discharge date. Definitions of medication use are provided in **Supplemental Table 2**.  † PCSK9mAbs were referred as proprotein convertase subtilisin/kexin type 9 inhibitors in the 2018 American Heart Association (AHA)/American College of Cardiology (ACC)/Multi-society guideline on the management of blood cholesterol.^10^  ‡ For patients with an LDL-C test within 30 days after their MI hospital discharge date, patient characteristics in the 30 days after their MI hospital discharge date were considered to be present only if these preceded or may have led to the LDL-C test. Specifically, we considered cardiologist care and primary care ambulatory visits to have occurred only if patients did not have an LDL-C test between their MI hospital discharge date and 3 days before their earliest cardiologist or primary care physician visits, respectively. We included outpatient cardiologist and primary care physician visits up to 3 days after an LDL-C test as this may have been ordered in advance of the visit. We considered re-hospitalization, recurrent MI, coronary revascularization procedure, and cardiac rehabilitation to have occurred only if patients did not have an LDL-C test between their MI hospital discharge date and the date of their earliest re-hospitalization, recurrent MI, coronary revascularization procedure, or cardiac rehabilitation session, respectively. | | |

## Supplemental Table 7.

Characteristic of patients with an MI hospitalization in the 5% fee-for-service cohort (secondary analysis)

|  | Patients with continuous pharmacy benefits from 365 days prior to through 30 days after their MI hospital discharge | |
| --- | --- | --- |
| Patient characteristics* | No  n=10,394 | Yes  n=21,690 |
| Calendar year of the MI hospital discharge, n (%) |  |  |
| 2016 | 2,513 (24.2) | 4,851 (22.4) |
| 2017 | 2,710 (26.1) | 5,621 (25.9) |
| 2018 | 2,708 (26.1) | 5,854 (27.0) |
| 2019 | 2,463 (23.7) | 5,364 (24.7) |
| Age, n (%) |  |  |
| 66 to 75 years | 4,818 (46.4) | 10,351 (47.7) |
| ≥76 years | 5,576 (53.6) | 11,339 (52.3) |
| Male, n (%) | 6,360 (61.2) | 11,224 (51.7) |
| Race/ethnicity, n (%) |  |  |
| Non-Hispanic white | 9,098 (87.5) | 18,438 (85.0) |
| Non-Hispanic Black | 802 (7.7) | 1,812 (8.4) |
| Asian | 82 (0.8) | 368 (1.7) |
| Hispanic | 64 (0.6) | 367 (1.7) |
| Other | 348 (3.3) | 705 (3.3) |
| Geographic region of residence, n (%) |  |  |
| New England | 569 (5.5) | 1,485 (6.8) |
| West South Central | 1,287 (12.4) | 2,408 (11.1) |
| Mountain | 655 (6.3) | 1,090 (5.0) |
| East South Central | 795 (7.6) | 1,668 (7.7) |
| Middle Atlantic | 1,194 (11.5) | 2,926 (13.5) |
| South Atlantic | 2,448 (23.6) | 4,426 (20.4) |
| West North Central | 736 (7.1) | 1,554 (7.2) |
| East North Central | 1,575 (15.2) | 3,650 (16.8) |
| Pacific | 1,135 (10.9) | 2,483 (11.4) |
| Diabetes, n (%) | 4,743 (45.6) | 11,347 (52.3) |
| Chronic kidney disease, n (%) | 5,969 (57.4) | 13,610 (62.7) |
| Heart failure, n (%) | 5,315 (51.1) | 12,115 (55.9) |
| History of stroke, n (%) | 1,278 (12.3) | 3,043 (14.0) |
| History of lower extremity artery disease, n (%) | 2,859 (27.5) | 6,979 (32.2) |
| Recent ACS, n (%) | 1,169 (11.2) | 2,927 (13.5) |
| History of CHD, n (%) | 6,697 (64.4) | 15,300 (70.5) |
| Coronary revascularization during the MI hospitalization, n (%) | 6,039 (58.1) | 11,972 (55.2) |
| **Patient characteristics defined using claims in the 30 days after each patient’s MI hospital discharge date†** | | |
| Cardiologist outpatient visits, n (%) | 3,896 (37.5) | 8,387 (38.7) |
| Primary care physician outpatient visits, n (%) | 4,801 (46.2) | 10,985 (50.6) |
| Re-hospitalization, n (%) | 1,307 (12.6) | 3,217 (14.8) |
| Recurrent MI, n (%) | 432 (4.2) | 1,089 (5.0) |
| Coronary revascularization procedure, n (%) | 2,245 (21.6) | 4,617 (21.3) |
| Cardiac rehabilitation, n (%) | 875 (8.4) | 1,742 (8.0) |
| ACS: acute coronary syndrome; CHD: coronary heart disease; MI: myocardial infarction; PCSK9mAb: proprotein convertase subtilisin/kexin type 9 monoclonal antibody.  * Dual Medicare-Medicaid eligibility/low-income subsidy for prescription medications and medication use were not measured as these may be underestimated among patients without pharmacy benefits.  † For patients with an LDL-C test within 30 days after their MI hospital discharge date, patient characteristics in the 30 days after their MI hospital discharge date were considered to be present only if these preceded or may have led to the LDL-C test. Specifically, we considered cardiologist care and primary care ambulatory visits to have occurred only if patients did not have an LDL-C test between their MI hospital discharge date and 3 days before their earliest cardiologist or primary care physician visits, respectively. We included outpatient cardiologist and primary care physician visits up to 3 days after an LDL-C test as this may have been ordered in advance of the visit. We considered re-hospitalization, recurrent MI, coronary revascularization procedure, and cardiac rehabilitation to have occurred only if patients did not have an LDL-C test between their MI hospital discharge date and the date of their earliest re-hospitalization, recurrent MI, coronary revascularization procedure, or cardiac rehabilitation session, respectively. | | |

## Supplemental Table 8.

Characteristic of patients with an MI hospitalization in the Optum’s de-identified Clinformatics® Data Mart Database (secondary analysis)

| Patient characteristics* | All MI hospitalizations  n=176,268 |
| --- | --- |
| Calendar year of the MI hospital discharge, n (%) |  |
| 2016 | 26,344 (15.0) |
| 2017 | 34,819 (19.8) |
| 2018 | 40,281 (22.9) |
| 2019 | 43,961 (24.9) |
| 2020 | 30,863 (17.5) |
| Age, n (%) |  |
| 66 to 75 years | 71,338 (40.5) |
| ≥76 years | 104,930 (59.5) |
| Male, n (%) | 90,946 (51.6) |
| Race/ethnicity, n (%) |  |
| Non-Hispanic white | 122,760 (69.6) |
| Non-Hispanic Black | 21,883 (12.4) |
| Asian | 4,282 (2.4) |
| Hispanic | 18,945 (10.8) |
| Other | 8,398 (4.8) |
| Geographic region of residence, n (%) |  |
| New England | 8,807 (5.0) |
| West South Central | 22,614 (12.8) |
| Mountain | 14,715 (8.4) |
| East South Central | 7,694 (4.4) |
| Middle Atlantic | 17,343 (9.8) |
| South Atlantic | 44,152 (25.1) |
| West North Central | 12,761 (7.2) |
| East North Central | 23,713 (13.5) |
| Pacific | 24,399 (13.8) |
| Diabetes, n (%) | 87,534 (49.7) |
| Chronic kidney disease, n (%) | 69,822 (39.6) |
| Heart failure, n (%) | 98,018 (55.6) |
| History of stroke, n (%) | 9,189 (5.2) |
| History of lower extremity artery disease, n (%) | 38,302 (21.7) |
| Recent ACS, n (%) | 18,885 (10.7) |
| History of CHD, n (%) | 47,256 (26.8) |
| Coronary revascularization during the MI hospitalization, n (%) | 74,836 (42.5) |
| **Medication use†** | |
| Statin use |  |
| Intensity, n (%) |  |
| None | 71,500 (40.6) |
| Low/Moderate-intensity therapy | 44,930 (25.5) |
| High-intensity therapy | 59,838 (34.0) |
| Initiation, n (%) | 30,106 (17.1) |
| Up-titration, n (%) | 9,148 (5.2) |
| Ezetimibe use, n (%) |  |
| Prevalent use | 3,203 (1.8) |
| Initiation | 685 (0.4) |
| PCSK9mAb† use, n (%) | 338 (0.2) |
| **Patient characteristics defined using claims in the 30 days after each patient’s MI hospital discharge date§** | |
| Cardiologist outpatient visits, n (%) | 35,901 (29.9) |
| Primary care physician outpatient visits, n (%) | 58,302 (48.5) |
| Re-hospitalization, n (%) | 32,954 (27.4) |
| Recurrent MI, n (%) | 12,852 (10.7) |
| Coronary revascularization procedure, n (%) | 7,644 (6.7) |
| Cardiac rehabilitation, n (%) | 7,998 (6.7) |
| ACS: acute coronary syndrome; CHD: coronary heart disease; MI: myocardial infarction; NA: not available; PCSK9mAb: proprotein convertase subtilisin/kexin type 9 monoclonal antibody.  * Data on Dual Medicare-Medicaid eligibility/Low-income subsidy for prescription medications were not available in the Optum’s de-identified Clinformatics® Data Mart Database. Indicators of patient comorbidities, including diabetes, chronic kidney disease, heart failure, history of stroke, and history of extremity artery disease, in the Optum’s de-identified Clinformatics® Data Mart Database were assessed using claims in the 365 days prior to each patient’s index date (i.e., each patient’s myocardial infarction hospital discharge date). Recent ACS and history of CHD were defined using claims in the 365 days prior to each patient’s myocardial infarction hospital admission date.  † Medication use was defined using pharmacy claims in the 90 days prior to each patient’s MI hospital admission date and within 7 days after each patient’s MI hospital discharge date. Definitions of medication use are provided in **Supplemental Table 2**.  ‡ PCSK9mAbs were referred as proprotein convertase subtilisin/kexin type 9 inhibitors in the 2018 American Heart Association (AHA)/American College of Cardiology (ACC)/Multi-society guideline on the management of blood cholesterol.^10^  § For patients with an LDL-C test within 30 days after their MI hospital discharge date, patient characteristics in the 30 days after their MI hospital discharge date were considered to be present only if these preceded the LDL-C test. | |

## Supplemental Figure 1.

Flow-chart of Medicare beneficiaries in the fee-for-service with pharmacy benefits cohort (primary analysis)

All beneficiaries with Medicare fee-for-service coverage and pharmacy benefits who had an MI hospitalization with a discharge date between January 1, 2016, and December 31, 2020, inclusive

MI hospitalizations: 1,423,407 (unique beneficiaries: 1,105,655)

Beneficiaries who were ≥66 years of age on their MI hospital discharge date

MI hospitalizations: 1,152,577 (unique beneficiaries: 953,770)

Beneficiaries who were discharged alive

MI hospitalizations: 952,641 (unique beneficiaries: 797,072)

Beneficiaries who were alive and had Medicare fee-for-service inpatient, outpatient, and pharmacy coverage for 30 days following their MI hospital discharge

MI hospitalizations: 823,261 (unique beneficiaries: 693,099)

Beneficiaries who had continuous fee-for-service inpatient, outpatient, and pharmacy coverage and were living in the US for the 365 days prior to their MI hospital discharge

MI hospitalizations: 1,050,220 (unique beneficiaries: 878,975)

Beneficiaries who were not admitted to a skilled nursing facility within 30 days following their MI hospital discharge

MI hospitalizations: 532,767 (unique beneficiaries: 465,553)

MI: myocardial infarction.

An MI hospitalization was defined by an inpatient claim with an overnight stay and less than 30 days of duration with an International Classification of Diseases, tenth revision code of I21.xxx or I22.xxx in any discharge diagnosis position.

## Supplemental Figure 2.

Flow-chart of Medicare beneficiaries in the 5% fee-for-service cohort (secondary analysis)

5% random sample of beneficiaries with Medicare fee-for-service coverage who had an MI hospitalization with a discharge date between January 1, 2016, and December 31, 2019, inclusive

MI hospitalizations: 108,591 (unique beneficiaries: 84,289)

Beneficiaries who were ≥66 years of age on their MI hospital discharge date

MI hospitalizations: 72,721 (unique beneficiaries: 62,182)

Beneficiaries who were discharged alive

MI hospitalizations: 57,635 (unique beneficiaries: 49,197)

Beneficiaries who were alive and had Medicare fee-for-service inpatient and outpatient coverage, with or without pharmacy coverage, for 30 days following their MI hospital discharge

MI hospitalizations: 49,677 (unique beneficiaries: 42,701)

Beneficiaries who had continuous fee-for-service inpatient and outpatient coverage, with or without pharmacy coverage, and were living in the US for the 365 days prior to their MI hospital discharge

MI hospitalizations: 63,514 (unique beneficiaries: 54,284)

Beneficiaries who were not admitted to a skilled nursing facility within 30 days following their MI hospital discharge

MI hospitalizations: 32,084 (unique beneficiaries: 28,507).

Beneficiaries with continuous pharmacy coverage from 365 days prior to through 30 days after their MI hospital discharge (i.e., those with continuous pharmacy benefits).

MI hospitalizations: 21,690.

Beneficiaries without continuous pharmacy coverage from 365 days prior to through 30 days after their MI hospital discharge (i.e., those without continuous pharmacy benefits).

MI hospitalizations: 10,394.

MI: myocardial infarction.

An MI hospitalization was defined by an inpatient claim with an overnight stay and less than 30 days of duration with an International Classification of Diseases, tenth revision code of I21.xxx or I22.xxx in any discharge diagnosis position.

## Supplemental Figure 3.

Beneficiaries in the Optum’s de-identified Clinformatics® Data Mart Database who had an MI hospitalization with a discharge date between January 1, 2016, and September 30, 2020, inclusive

MI hospitalizations: 387,149 (unique beneficiaries: 339,274)

Beneficiaries who had continuous Medicare Advantage coverage and were living in the US for the 365 days prior to their MI hospital discharge

MI hospitalizations: 252,811 (unique beneficiaries: 219,288)

Beneficiaries who were discharged alive

MI hospitalizations: 228,104 (unique beneficiaries: 198,794)

Beneficiaries who were ≥66 years of age on their MI hospital discharge date

MI hospitalizations: 228,291 (unique beneficiaries: 198,960)

Beneficiaries who were alive and had Medicare Advantage coverage for 30 days following their MI hospital discharge

MI hospitalizations: 176,268 (unique beneficiaries: 155,348)

Flow-chart of beneficiaries with Medicare Advantage coverage in the Optum’s de-identified Clinformatics® Data Mart Database (secondary analysis)

MI: myocardial infarction.

An MI hospitalization was defined by an inpatient claim with an overnight stay and less than 30 days of duration with an International Classification of Diseases, tenth revision code of I21.xxx or I22.xxx in any discharge diagnosis position.

## Supplemental Figure 4.

Schematic of the study design

Examine patient characteristics after the MI hospital discharge

-365 days

Examine patient characteristics during the MI hospitalization

MI hospital discharge between January 1, 2016, and December 31, 2020

Assessment of LDL-C testing

Patient timeline

MI admission date

Days prior to the MI hospital discharge

Days after the MI hospital discharge

365 days

30 days

Examine patient characteristics before the MI hospitalization

Period on which the patient was alive and had continuous fee-for-service Medicare coverage with pharmacy benefits.

LDL-C: low-density lipoprotein cholesterol; MI: myocardial infarction.

## Supplemental Figure 5.

Cumulative incidence of a lipid test following hospital discharge for MI in the fee-for-service with pharmacy benefits cohort (sensitivity analysis)


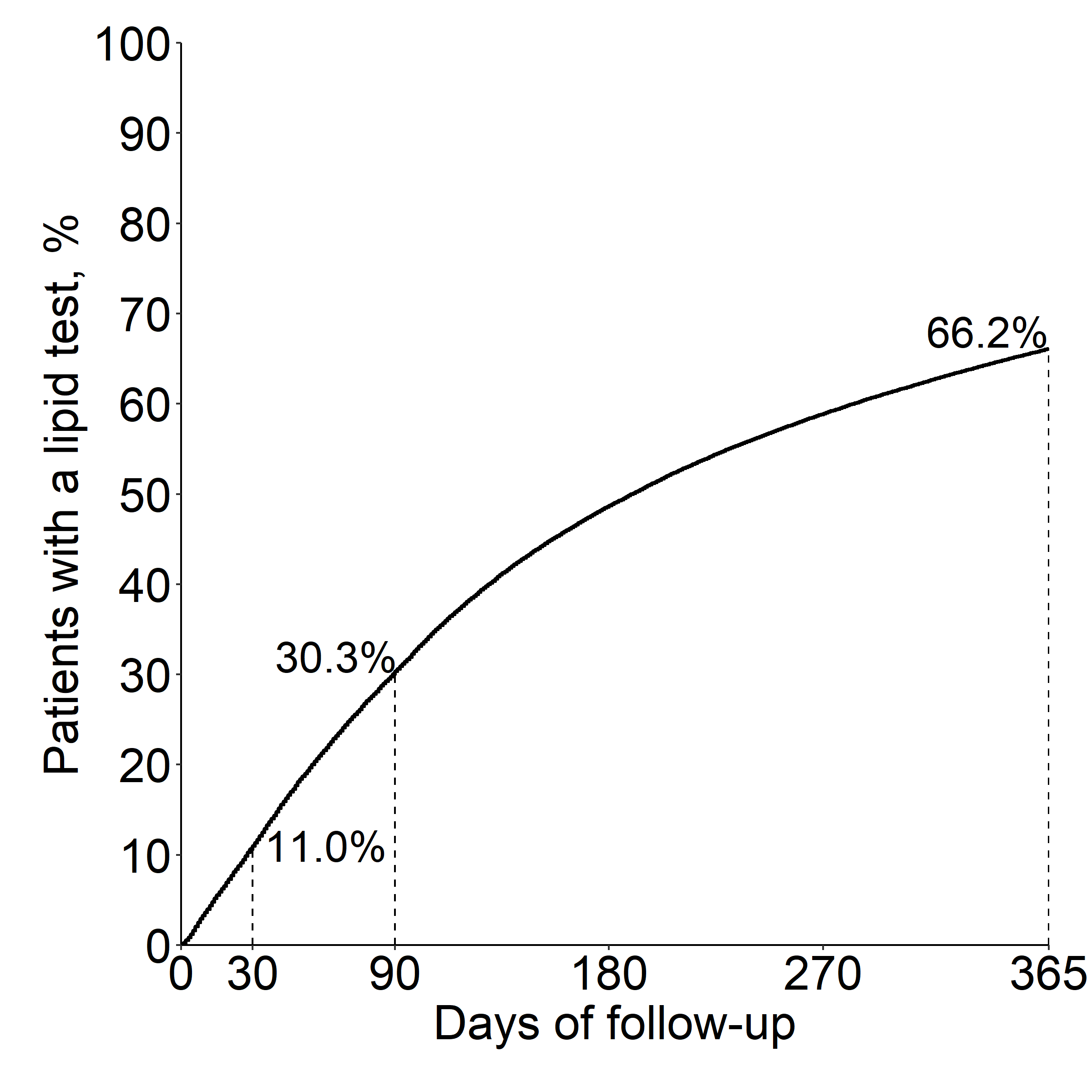


MI: myocardial infarction.

The definition of a lipid test includes current procedural terminology code for LDL-C tests (i.e., 80061, 83721) and other lipid tests which may indicate that LDL-C was known (see **Supplemental Table 1**).

## Supplemental Figure 6.

Cumulative incidence of an LDL-C test following hospital discharge for MI in the fee-for-service with pharmacy benefits cohort (sensitivity analysis)


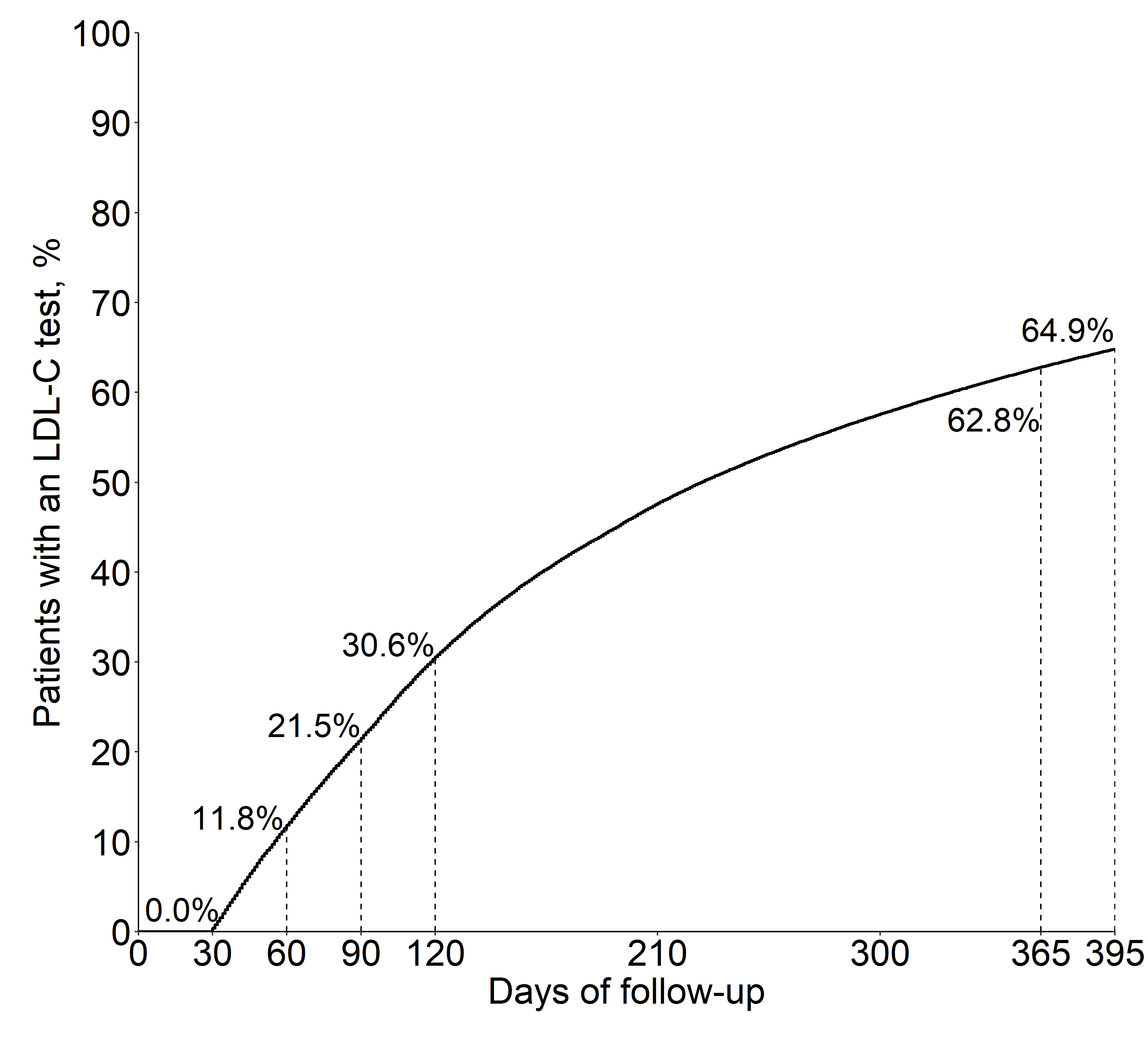


LDL-C: low-density lipoprotein cholesterol; MI: myocardial infarction.

The definition of LDL-C test includes an outpatient claim with a current procedural terminology code of 83721 or 80061.

## Supplemental Figure 7.

Cumulative incidence of an LDL-C test following hospital discharge for MI in the 5% fee-for-service cohort (secondary analysis)


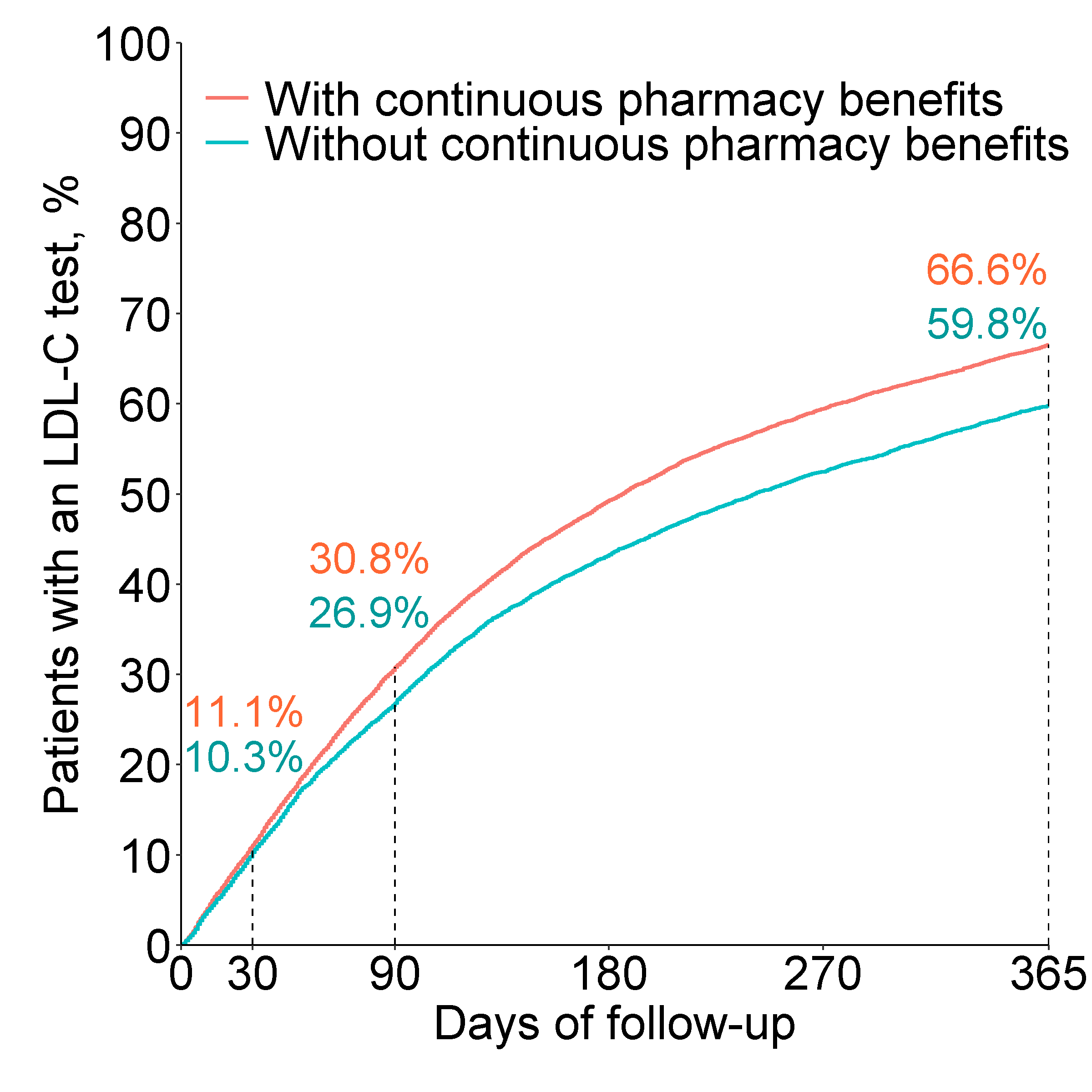


LDL-C: low-density lipoprotein cholesterol; MI: myocardial infarction.

The definition of LDL-C test includes an outpatient claim with a current procedural terminology code of 83721 or 80061.

Continuous pharmacy benefits was defined by having pharmacy coverage from 365 days prior to through 30 days after each patient’s MI hospital discharge date.

## References

1. Elixhauser A, Steiner C, Harris DR, Coffey RM. Comorbidity measures for use with administrative data. Med Care. 1998;36(1):8-27.

2. Gandra SR, Lawrence LW, Parasuraman BM, Darin RM, Sherman JJ, Wall JL. Total and component health care costs in a non-Medicare HMO population of patients with and without type 2 diabetes and with and without macrovascular disease. J Manag Care Pharm. 2006;12(7):546-554.

3. Quan H, Li B, Saunders LD, Parsons GA, Nilsson CI, Alibhai A, Ghali WA. Assessing validity of ICD-9-CM and ICD-10 administrative data in recording clinical conditions in a unique dually coded database. Health Serv Res. 2008;43(4):1424-1441.

4. Muntner P, Gutierrez OM, Zhao H, Fox CS, Wright NC, Curtis JR, McClellan W, Wang H, Kilgore M, Warnock DG, Bowling CB. Validation study of medicare claims to identify older US adults with CKD using the Reasons for Geographic and Racial Differences in Stroke (REGARDS) Study. Am J Kidney Dis. 2015;65(2):249-258.

5. Grams ME, Plantinga LC, Hedgeman E, Saran R, Myers GL, Williams DE, Powe NR. Validation of CKD and related conditions in existing data sets: A systematic review. Am J Kidney Dis. 2011;57(1):44-54.

6. Schneider KM, O'Donnell BE, Dean D. Prevalence of multiple chronic conditions in the United States' Medicare population. Health Qual Life Outcomes. 2009;7:82.

7. Kumamaru H, Judd SE, Curtis JR, Ramachandran R, Hardy NC, Rhodes JD, Safford MM, Kissela BM, Howard G, Jalbert JJ, Brott TG, Setoguchi S. Validity of claims-based stroke algorithms in contemporary Medicare data: REasons for Geographic And Racial Differences in Stroke (REGARDS) study linked with medicare claims. Circ Cardiovasc Qual Outcomes. 2014;7(4):611-619.

8. Colantonio LD, Hubbard D, Monda KL, Mues KE, Huang L, Dai Y, Jackson EA, Brown TM, Rosenson RS, Woodward M, Muntner P, Farkouh ME. Atherosclerotic Risk and Statin Use Among Patients With Peripheral Artery Disease. J Am Coll Cardiol. 2020;76(3):251-264.

9. Kent ST, Safford MM, Zhao H, Levitan EB, Curtis JR, Kilpatrick RD, Kilgore ML, Muntner P. Optimal Use of Available Claims to Identify a Medicare Population Free of Coronary Heart Disease. Am J Epidemiol. 2015;182(9):808-819.

10. Grundy SM, Stone NJ, Bailey AL, Beam C, Birtcher KK, Blumenthal RS, Braun LT, de Ferranti S, Faiella-Tommasino J, Forman DE, Goldberg R, Heidenreich PA, Hlatky MA, Jones DW, Lloyd-Jones D, Lopez-Pajares N, Ndumele CE, Orringer CE, Peralta CA, Saseen JJ, Smith SC, Jr., Sperling L, Virani SS, Yeboah J. 2018 AHA/ACC/AACVPR/AAPA/ABC/ACPM/ADA/AGS/APhA/ASPC/NLA/PCNA Guideline on the Management of Blood Cholesterol: A Report of the American College of Cardiology/American Heart Association Task Force on Clinical Practice Guidelines. J Am Coll Cardiol. 2019;73(24):e285-e350.
